# Evaluating the Performance and Repeatability of Poroelastic and Poroviscoelastic Models in Intrinsic MR Elastography

**DOI:** 10.1002/nbm.70073

**Published:** 2025-06-06

**Authors:** Marius Burman Ingeberg, Elijah Van Houten, Jaco J. M. Zwanenburg

**Affiliations:** ^1^ Translational Neuroimaging Group, Center for Image Sciences University Medical Center Utrecht Utrecht the Netherlands; ^2^ Department of Mechanical Engineering Université de Sherbrooke Sherbrooke Canada

**Keywords:** brain mechanics, cerebral vascular pulsations, DENSE, elastography, iMRE, poroelastic, poroviscoelastic

## Abstract

Intrinsic MR elastography (iMRE) leverages brain pulsations that arise from cerebral arterial pulsations to reconstruct the mechanical properties of the brain. While iMRE has shown much potential recently, the technique was demonstrated for a viscoelastic brain model only, which suffered from data‐model mismatch at the low actuation frequencies of the arterial pulsations. This work aims to address those limitations by considering the porous nature of brain tissue, where both a poroelastic and a poroviscoelastic model are assessed and compared. As a secondary goal, the influence of two driving frequencies on the material properties is investigated by looking at the 1 Hz and 2 Hz components of the motion data. The poroelastic and poroviscoelastic properties of the brain were reconstructed using a subzone‐based nonlinear inversion scheme, using displacement measurements of eight healthy subjects from a previous study at 7 T MRI. The performance of each model was evaluated by assessing consistency of spatial distributions, repeatability through repeated scans, and left–right symmetry. The poroelastic model yielded mean storage moduli of 6.08 ± 0.87 and 32.01 ± 11.92 Pa, and the poroviscoelastic model yielded 5.32 ± 0.87 and 26.15 ± 8.02 Pa for the 1‐ and 2‐Hz motion components, respectively. Among the mechanical properties of interest, the storage modulus was the most consistent, with low limits of agreement of (upper/lower) 15.0%/−22.2% for the poroelastic model and 10.9%/−18.5% for the poroviscoelastic model, relative to the whole‐brain mean. It was also highly symmetric, with a mean whole‐brain symmetry ratio of 0.99 across subjects for both models. Mechanical properties related to fluid flow demonstrated less consistency. The 2‐Hz motion component was found to contain considerable information as it reflected the frequency‐related stiffening associated with porous media, highlighting its relevance for use in multifrequency mechanical characterization. Both models demonstrated good performance, with the poroviscoelastic model in general showing the highest consistency in terms of test–retest repeatability. Future work aims to improve the models by addressing current assumptions on the boundary conditions of the pressure field.

## Graphical abstract

Poroelastic and poroviscoelastic property reconstruction is found to be feasible using intrinsic MR elastography. The poroviscoelastic model was found to be more suitable for intrinsic magnetic resonance elastography (MRE) elastography in the current implementation. The brain exhibits ultra‐soft properties at the low driving frequencies given by the cardiac pulse.

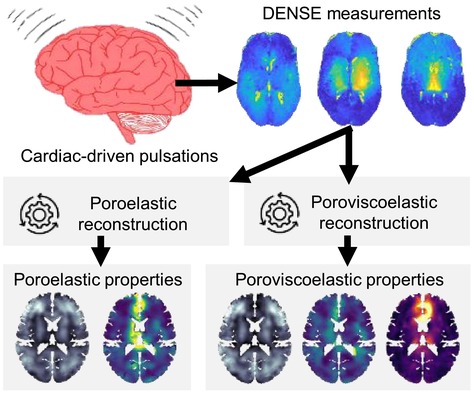

**Abbreviations**
CGM: cortical gray matter
CSF: cerebrospinal fluid
DENSE: displacement encoding with stimulated echoes
FFT: fast Fourier transform
iMRE: intrinsic magnetic resonance elastography
LoA: limits of agreement
MRE: magnetic resonance elastography
SGM: subcortical gray matter
WMT: white matter tracts

## 1 Introduction

Magnetic resonance elastography (MRE) is an advanced imaging technique that enables noninvasive in vivo measurements of tissue mechanical properties, providing insight into how different pathologies affect tissue microstructure [1, 2, 3, 4]. While it can in principle be applied to any tissue, there has recently been much interest in its potential for assessing brain pathology such as tumors [5], neurodegenerative diseases [6], and small vessel disease [7]. Furthermore, it may also provide insight into the relationship between brain structure and function [8, 9, 10, 11, 12], all while the brain is its natural environment. The standard approach uses mechanical actuators, typically pneumatic pillows positioned beneath the subject's head, to generate steady‐state mechanical waves within the region of interest. These waves induce displacement fields that can be captured using phase‐contrast imaging techniques, which are subsequently processed using various inversion algorithms to reconstruct the tissue's mechanical properties [13].

Recent work has demonstrated the feasibility of a technique called intrinsic MRE (iMRE), which eliminates the need for external actuation by leveraging the naturally occurring cerebral vascular pulsations driven by the cardiac pulse [14, 15, 16, 17]. iMRE is particularly advantageous for a few reasons: It eliminates the requirement for external actuators, thereby simplifying the acquisition process and significantly enhancing the method's accessibility; it addresses the challenge of poor shear wave penetration through the skull [16], and it provides insights into the brain's mechanical properties in its natural state, rather than in a state of externally generated vibration. However, despite the demonstrated feasibility of the method, a number of challenges were also highlighted, such as the occurrence of artifacts due to fluid motion present within the brain volume, the recovery of nonunique solutions when using a viscoelastic model at low actuation frequencies, and data‐model mismatch. On the other hand, the adoption of a poroelastic model, which models the brain as a porous elastic matrix that is saturated by a viscous pore fluid, has been shown to mitigate many of these issues (M. [18]; M. D. J. [19, 20]). Specifically, a poroelastic model can facilitate the recovery of unique solutions and provide a more accurate representation of the brain's behavior at low actuation frequencies. Moreover, poroelastic reconstruction enables the estimation of the hydraulic permeability, an additional parameter that most likely reflects microvascular and/or interstitial fluid properties. This could be highly relevant for understanding cerebrovascular diseases and the impact of vascular dysfunction in neurodegenerative disorders, highlighting another potential advantage of iMRE as the suitability of poroelastic models is less evident at higher actuation frequencies (M. [18]; M. D. J. [19]).

While the advantages of employing a poroelastic model at low actuation frequencies have been previously shown (M. [18]; M. D. J. [19]), thorough analysis of the performance of such a model has not been conducted. Furthermore, a poroelastic model can be extended to a poroviscoelastic model which additionally accounts for the viscous properties of the tissue at the cost of additional model complexity. It is currently unknown which model is best suited to recover accurate representations of the underlying tissue properties. The aim of this work is thus to investigate the performance and reliability of employing poroelastic and poroviscoelastic models using iMRE and to directly compare results between the two models. This is assessed by investigating regional variation, repeatability (by the means of repeated scans), and right–left symmetry over different regions of interest. As a secondary goal, we investigate how the material properties are influenced by the driving frequencies by looking at the 1‐ and 2‐Hz components of the motion data in the frequency domain, for both models. This analysis serves several purposes: first, to evaluate the impact of different low‐frequency driving conditions on material properties and second, to examine how noisier data (given the lower signal to noise ratio within the 2‐Hz cardiac component) affect the quality of mechanical property reconstruction. This enables us to test the robustness of the models under varying noise levels and frequency ranges, ultimately helping to determine the feasibility of incorporating higher‐frequency components in multifrequency reconstructions within iMRE.

## 2 Method

### 2.1 Displacement Data

This following analysis was conducted on pre‐existing displacement data, acquired by Adams et al. [21]. We only briefly summarize the method of acquisition here, as a detailed description can be found in the original publication. All subjects gave written informed consent for participation in the study, which was approved by the ethical review board of our institution.

Eight healthy young adults (three females; mean age: 27 ± 6 years) underwent imaging using a displacement encoding with stimulated echoes (DENSE) sequence on a 7‐T MR scanner (Philips Healthcare) using a 3D EPI readout scheme. Repeated scans were performed with subjects briefly exiting the scanner for up to 10 min between sessions. Retrospective gating was used to synchronize DENSE measurements with the cardiac cycle, detected via pulse oximetry. The acquired spatial resolution was 1.95 mm × 1.95 mm × 2.2 mm (AP, RL, FH), with 20 temporal phases reconstructed per cardiac cycle. Three separate acquisitions were performed with displacement encoding (DENC) sensitivities of 0.175, 0.175, and 0.35 mm in the anterior–posterior (AP), right–left (RL), and foot‐head (FH) directions, respectively. The acquisition time per motion encoding direction was 144 heartbeats (which correspond to 2:24 min for an average heart rate of 60 beats per minute). Additionally, T1‐weighted images with a resolution of 0.93 mm × 0.93 mm × 1.0 mm were acquired during both scanning sessions for segmentation and registration purposes. The Computational Anatomy Toolbox (Jena University Hospital, Departments of Psychiatry and Neurology) extension for SPM12 (Wellcome Trust Centre for Neuroimaging, University College London) was used to generate tissue probability maps from the T1‐weighted images. To account for subject motion between the three DENSE acquisitions, AP and FH magnitude images were rigidly registered to the RL magnitude images. The final RL image of the cardiac cycle was then nonlinearly registered to a T1‐weighted image for all subjects, as those images showed minimal artifacts and had the most optimal gray and white matter contrast. All images were then transformed to the T1‐weighted image using the registration parameters given by the transformation from the RL magnitude image to the T1‐weighted image.

The SNR of the displacement data was assessed as described by Adams et al. [21] and was used to identify voxels with motion artifacts. The number of voxels with motion artifacts was used as a metric to evaluate the quality of each data set. One repeated scan of Subject 7 was omitted due to containing more than twice as many voxels with motion artifacts in the displacement measurements as compared to the mean of all other scans.

### 2.2 Preparation of Displacement Data

**FIGURE 1:**
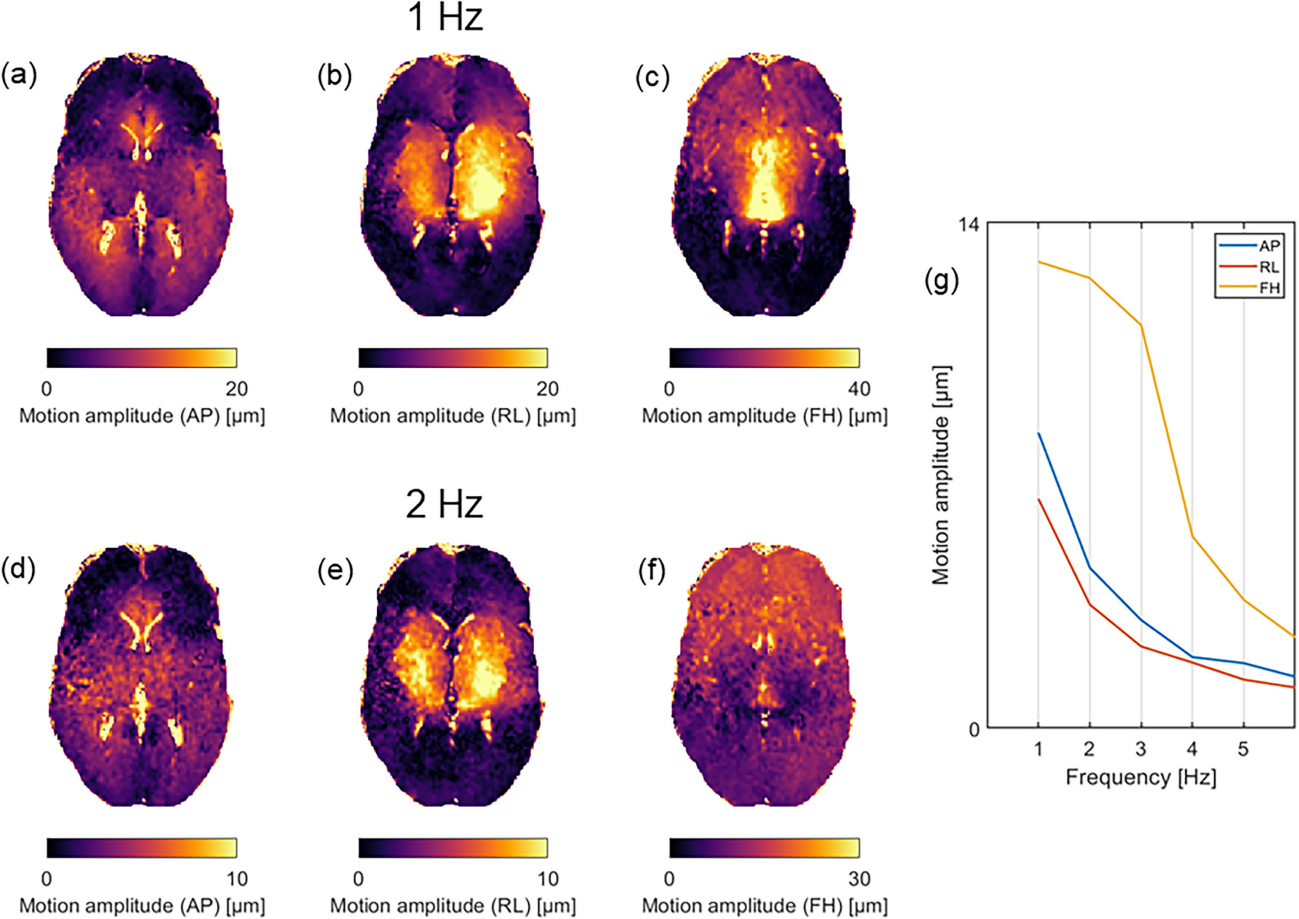
Representative axial slices of motion amplitude fields for each motion encoding direction at 1 Hz (a–c) and 2 Hz (d–f) for a single subject. The mean motion amplitude across motion encoding directions is shown in (g) for the first five frequencies.

The displacement data were transformed into frequency space by applying the fast Fourier transform (FFT) to the temporal phases for each voxel. The motions at the first two harmonic frequencies, which roughly correspond to 1 and 2 Hz, were selected for further analysis. While there were some variations in the exact frequency across subjects due to varying heart rates, we use the names “1 Hz” and “2 Hz” for simplicity and clarity throughout the analysis. Figure 1 presents axial slices of the displacement amplitude fields for each motion encoding direction and frequency component along with the mean displacement amplitude for the first five frequency components. To address fluid motion artifacts from cerebrospinal fluid (CSF) and larger blood vessels, a fluid motion mask was generated for each subject, as reported in our previous iMRE study [14]. These masks were constructed by combining CSF masks derived from tissue probability maps—where regions with less than 95% probability of being gray or white matter were classified as CSF—and masks taking extreme displacements likely due to fluid motion. The threshold for extreme displacements was set at five times the standard deviation of the displacement distribution within the brain, determined empirically through trial runs across multiple subjects. To ensure minimal contribution from fluid motion in the displacement data, these masks were further dilated by one voxel.

Because mechanical property reconstruction relies on finite element meshes, gaps in the displacement data caused by fluid motion masking create large holes in the meshes, leading to significant issues. To address this, the gaps were interpolated using a publicly available MATLAB extension (John [22]) to ensure that displacements in these regions were continuous and differentiable. Following reconstruction, the fluid motion masks were reapplied to exclude all voxels where interpolated displacements were used, ensuring that the final parameter maps contained only values derived from actual measurements.

### 2.3 Mechanical Property Estimation

Tetrahedral finite element models of each subject's brain were created from the corresponding displacement maps. The meshes were constructed to have an average edge length of approximately 2 mm. These finite element models were then utilized in a subzone‐based nonlinear inversion scheme (M. D. J. [23, 24]) to estimate mechanical properties, using two brain models: a poroelastic model and a poroviscoelastic model. In a poroelastic model, the brain is modeled as a biphasic material consisting of a porous elastic matrix which is saturated by a viscous pore fluid. The mathematical details of such models have been extensively described in previous work (M. [18, 20, 25, 26]), and only the main features will be highlighted here. The equation of motion in the frequency domain is given by the following:

(1) $$\begin{array}{c} \nabla \cdot \mu \nabla u+\nabla \left(\lambda +\mu \right)\left(\nabla \cdot u\right)-\left(1-\beta \right)\nabla P=-\left(\rho -\beta \rho_{f}\right)\omega^{2}u\\ \rho_{f}\omega^{2}\nabla \cdot \left(\left(1-\beta \right)u\right)+\nabla \cdot \left(\beta \nabla P\right)=0\\ \;\beta =\frac{\omega \phi^{2}\rho_{f}\kappa }{\omega \kappa \left(\rho_{a}+\phi \rho_{f}\right)+i\phi^{2}}, \end{array}$$

where u is the 3D complex‐valued motion amplitude, $G^{\prime }$ and $\nu_{p}$ are the storage modulus and Poisson ratio of the drained porous solid, κ is the hydraulic permeability (sometimes reported as hydraulic conductivity in literature), P is the pore fluid pressure, $\rho_{f}$ is the pore fluid density, $\rho_{a}$ is the apparent mass density, and ϕ is the relative porosity. Lamé's first constant (here referred to as the lambda modulus) is defined by the following:

$$\lambda =\frac{2G^{\prime }\nu_{p}}{1-2\nu_{p}},$$

and describes the compressive resistance of the drained porous solid. While the model assumes the individual material constituents (i.e., the solid and fluid components) to be incompressible, the mechanical properties of the drained solid matrix must be compressible to allow for fluid motion in response to volumetric strain [25]. Thus, instead of setting λ to a large value to model incompressibility, as is typically done in viscoelastic elastography, it becomes a property of interest as it describes the resistance of the tissue matrix to volumetric changes in the absence of vascular and/or extracellular fluids.

The formulation shown in Equation (1) is based on several assumptions, including time‐harmonic displacements, the absence of fluid sources, and the incompressibility of the constituent solid and fluid components. The poroviscoelastic model is identical to the abovementioned formulation, with the exception that the storage modulus is replaced by the complex‐valued shear modulus $G^{*}=G^{\prime }+{\mathrm{iG}}^{\prime \prime }$, where the real part is the storage modulus and the imaginary part is the loss modulus, representing the viscosity of the drained solid matrix. Using a poroelastic model, three properties of interest are recovered: The storage modulus $G^{\prime }$, Lamé's first constant λ, and the hydraulic permeability κ. The poroviscoelastic model recovers the same properties, with the addition of the loss modulus $G^{\prime }$, which can be used to calculate the damping ratio, $\xi =G^{\prime \prime }/2G^{\prime }$.

Poroelastic and poroviscoelastic property maps were reconstructed for the 1‐ and 2‐Hz displacement components for all subjects and repeated scans. A total of 600 global iterations were allowed for each subject, and a subzone size of 20 mm^3^ with 15% overlap between zones was used.

### 2.4 Regional Analysis

Differences in regional variation in the property maps between models and driving frequencies were assessed by performing a regional analysis. To enable direct comparison with existing literature, we analyzed the same regions as those used in the publicly available atlas (in standard‐space) of viscoelastic properties obtained with extrinsic actuation [27], as well as those investigated previously with iMRE using a viscoelastic brain model [14]. A total of 30 regions were investigated, categorized as cortical gray matter (CGM), subcortical gray matter (SGM), and white matter tracts (WMT). The CGM and SGM regions were identified using the MNI‐ICBM2009c nonlinear symmetric 1‐mm atlas [28], while the WMT regions were identified using the JHU‐ICBM‐tracts and JHU‐ICBM‐labels 1‐mm atlases [29, 30]. Each region mask was individually eroded by 1 voxel to minimize partial volume effects. Regions with less than 500 voxels after erosion were removed from the analysis. All included regions are presented in Table 1 with the corresponding major region categorization, abbreviation, and region size.

**TABLE 1:** Regions of interests with their corresponding major region categorization, abbreviation, and region size in number of voxels.

Major region	Minor region	Abbreviation	Region size
CGM	Cuneus	CN	2769
	Fusiform gyrus	FSG	5731
	Inferior temporal cortex	ITC	6444
	Lateral occipital cortex	LaO	10,660
	Lingual occipital cortex	LiO	5497
	Precuneus	PCN	9513
	Postcentral cortex	POST	2545
	Superior frontal cortex	SFC	24,885
	Superior parietal cortex	SPC	3298
	Superior temporal cortex	STC	9251
	Rostral middle frontal cortex	RMF	9037
	Precentral cortex	PRE	5682
SGM	Amygdala	AM	858
	Caudate	CA	3533
	Hippocampus	HC	2446
	Pallidum	PA	1067
	Putamen	PU	6240
	Thalamus	TH	10,112
WMT	Forceps major	FMa	1283
	Forceps minor	FMi	10,048
	Corpus callosum	CC	16,116
	Anterior thalamic radiation	ATR	6119
	Corticospinal tract	CST	2853
	Inferior frontal occipital fasciculus	IFOF	1656
	Inferior longitudinal fasciculus	ILF	1678
	Superior longitudinal fasciculus	SLF	3605
	Posterior thalamic radiation	PTR	19,957
	Corona radiata	CRa	5731

To determine the regional values of each property, all property maps were registered to MNI space using the MNI‐ICBM2009c nonlinear average template. To achieve this, the T1‐weighted images (which are in the same space as the property maps) of each subject were nonlinearly registered to the MNI template for two levels of resolutions with 250 iterations per resolution. This was done following an initial alignment using rigid registration for 100 iterations for four levels of resolution and affine registration for 100 iterations for two levels of resolution. The corresponding transformations were then applied to all property maps using nearest neighbor interpolation. The JHU‐ICBM atlases were additionally interpolated (nearest neighbor) to the resolution of the MNI‐ICBM2009c template to assure equivalent resolution of all atlases. Repeated measurements were averaged for all properties.

### 2.5 Repeatability Analysis

The repeatability of the poroelastic and poroviscoelastic brain models was assessed for the 1‐ and 2‐Hz displacement components across all subjects by performing a subject‐wise Bland–Altman analysis on the mean whole‐brain material properties. Considering the dependence of material properties on actuation frequency [31], the reported limits of agreement (LoA) were normalized to the mean global value to facilitate comparison with literature.

### 2.6 Symmetry Analysis

The left–right symmetry of the material property maps was investigated for the poroelastic and poroviscoelastic models for the 1‐ and 2‐Hz displacement components. This was done by computing the symmetry ratio s that we define as

$$s=\frac{p_{\text{mean},\text{right}}}{p_{\text{mean},\text{left}}},$$

where $p_{\text{mean},\text{right}}$ is the mean property value in the right hemisphere and $p_{\text{mean},\text{left}}$ is the mean property value of the left hemisphere. The material properties were averaged across repeated scans. The symmetry ratio was computed across all major brain regions and material properties.

### 2.7 Influence of Hydraulic Permeability

The influence of varying levels of hydraulic permeability on the storage modulus was investigated for one subject. This investigation was conducted for the purely poroelastic model and the 1‐Hz motion component. The estimation of mechanical properties followed the procedure outlined in Section 2.3, with the exception that hydraulic permeability was set to a fixed, homogeneous value across the brain. The permeability values investigated ranged from $\kappa =10^{-8}$ to $\kappa =10^{-4}$ m^3^s kg^−1^, evaluated at each order of magnitude.

## 3 Results

### 3.1 Representative Parameter Maps

**FIGURE 2:**
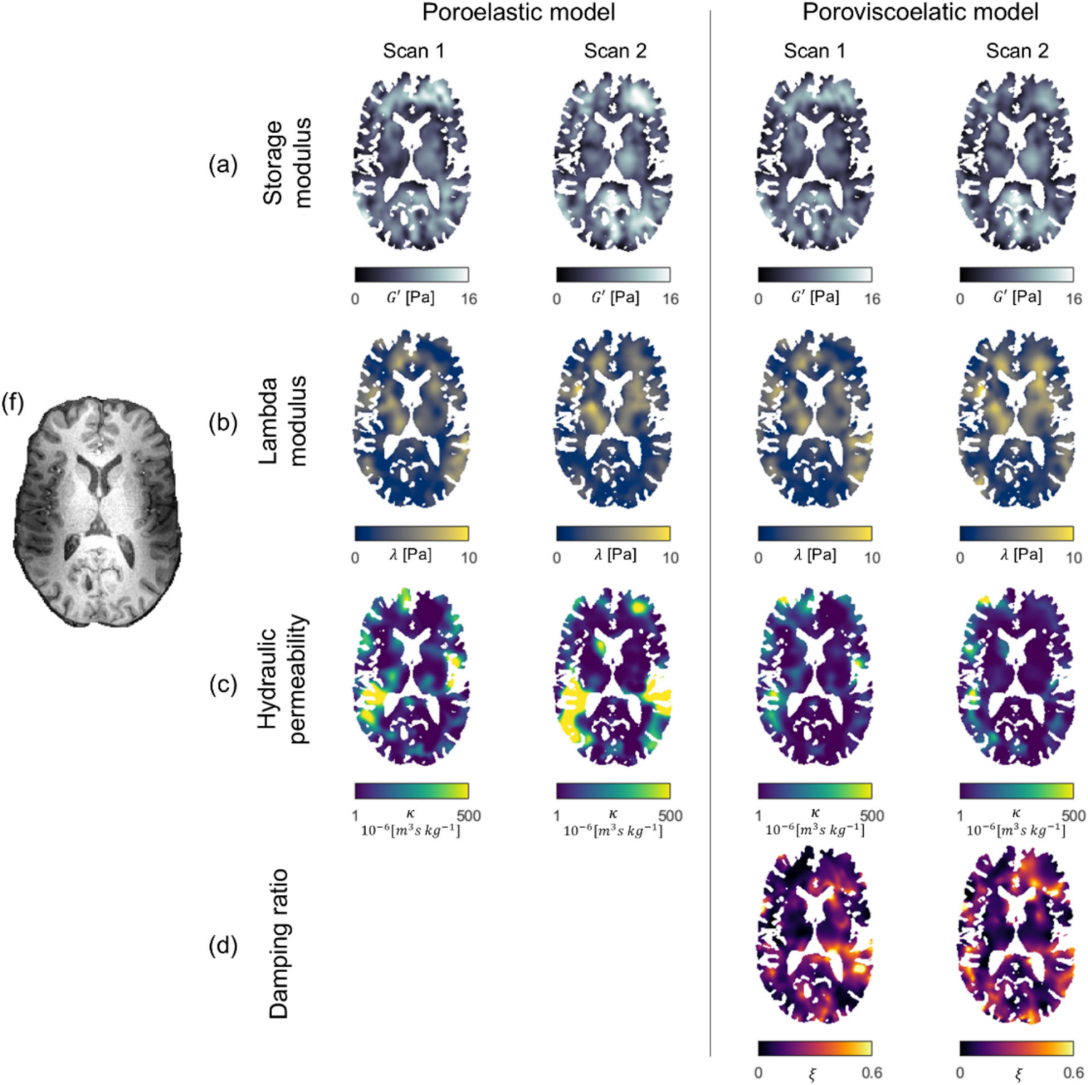
Representative axial slices of the storage modulus (a), lambda modulus (b), hydraulic permeability (c), and damping ratio (d) obtained from the 1‐Hz motion component for one subject along with a repeated scan which has been co‐registered to the first scan. The equivalent slice of the T1‐weighted image is shown in (f) for anatomical reference. All property maps show consistent spatial patterns across both repeated scans and material models.

The poroelastic and poroviscoelastic property maps were successfully reconstructed for all subjects, for both the 1‐ and 2‐Hz motion components. The tetrahedral finite element meshes consisted of (mean ± STD) 156,220 ± 8276 nodes. All property maps reached convergence, as expressed by a low total change in property values between the final iterations of the nonlinear inversion, which was less than 0.02% for all scans. Figure 2 displays representative axial slices for one subject of all property maps for both the poroelastic and poroviscoelastic models. The repeated scans are additionally placed alongside the original scans, where they have been co‐registered to the original scan using nonlinear registration of their respective T1‐weighted images. Similarity in spatial patterns can be seen between both repeated scans and material models across all properties. The largest differences between the models were observed in the hydraulic permeability maps, where the viscous attenuation in the poroviscoelastic model, expressed in terms of the damping ratio, seemed to be partly represented in the hydraulic permeability maps of the poroelastic model. No fluid motion‐induced artifacts were observed in the parameter maps. The storage modulus maps exhibited good left–right symmetry, while asymmetric low‐valued regions were observed in the hydraulic permeability maps. Figure S1 shows the equivalent figure for the 2‐Hz motion component property maps.

### 3.2 Group‐Wise Mean Maps

**FIGURE 3:**
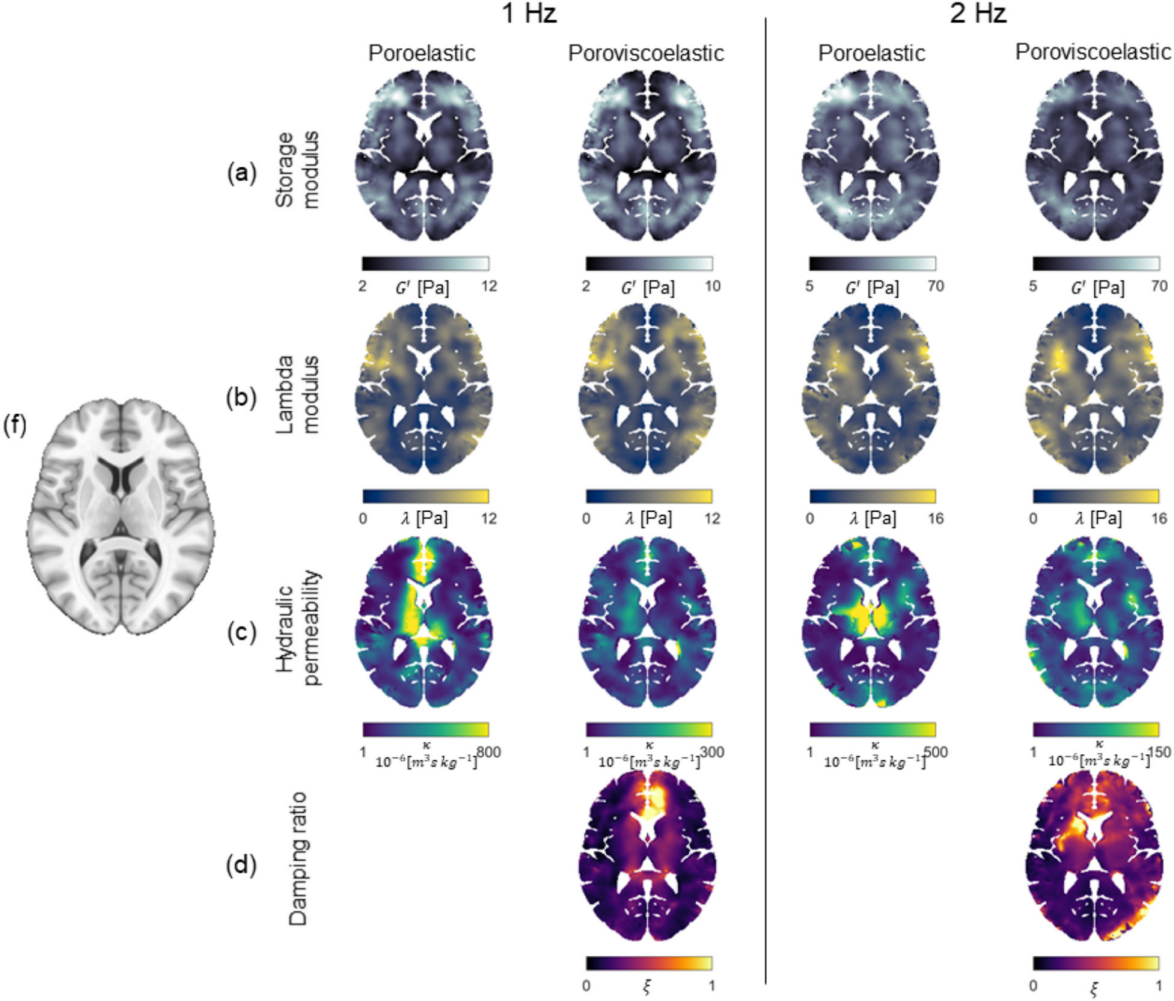
Axial slices of the mean storage modulus (a), lambda modulus (b), hydraulic permeability (c), and damping ratio (d) across all scans, where each property map has been registered to the MNI‐ICBM2009c nonlinear average template. The equivalent slice of the T1‐weighted template is shown in (f) for anatomical reference. The spatial patterns of the storage and lambda moduli are largely consistent across brain models, with slight variations observed between different motion components. The storage modulus and damping ratio exhibit good left–right symmetry, while the lambda modulus and hydraulic permeability exhibit poorer symmetry.

Figure 3 displays axial slices of maps containing the mean over all scans for all material properties of the poroelastic and poroviscoelastic brain models for both the 1‐ and 2‐Hz motion components. The spatial patterns of the storage and lambda modulus were substantially similar across brain models but varied slightly across motion components. Similar spatial patterns were observed in the poroelastic hydraulic permeability map as in the poroviscoelastic damping ratio map. The hydraulic permeability generally demonstrated lower values when using the poroviscoelastic model compared to the poroelastic model. The storage modulus maps exhibited excellent left–right symmetry in comparison to the other maps. The lambda modulus and hydraulic permeability maps showed less consistent symmetry.

### 3.3 Regional Analysis

**FIGURE 4:**
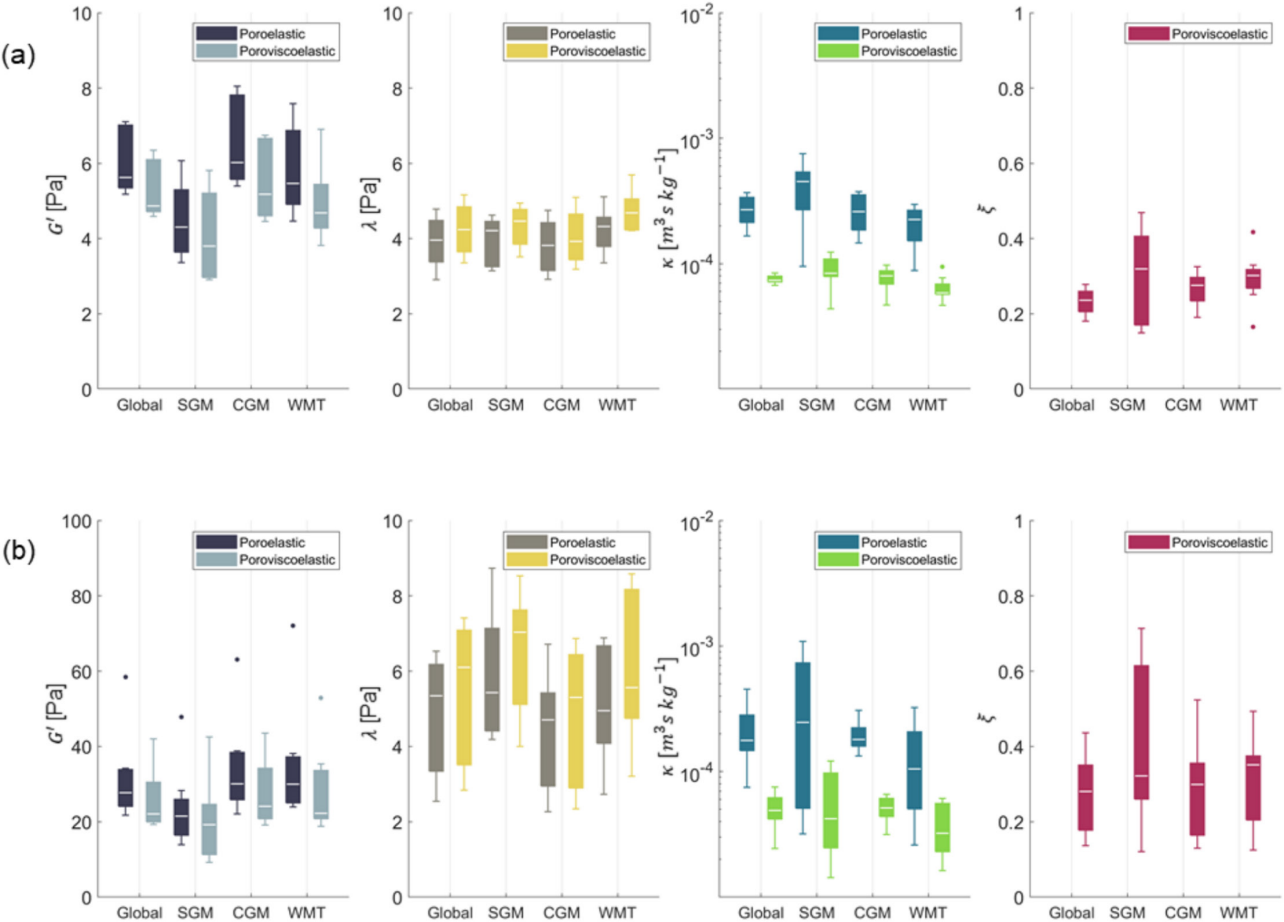
Boxplots illustrating the mean regional material properties across major regions for all subjects for the 1‐Hz motion component in (a) and 2‐Hz motion component in (b). The white line inside the boxplots represents the median value, while the upper and lower ends of the boxplots correspond to the 25th and 75th quartiles. The whiskers represent the maximum and minimum values which are not outliers (presented as dots and are defined as values that lie more than 1.5·IQR away from the upper or lower quartiles).

**FIGURE 5:**
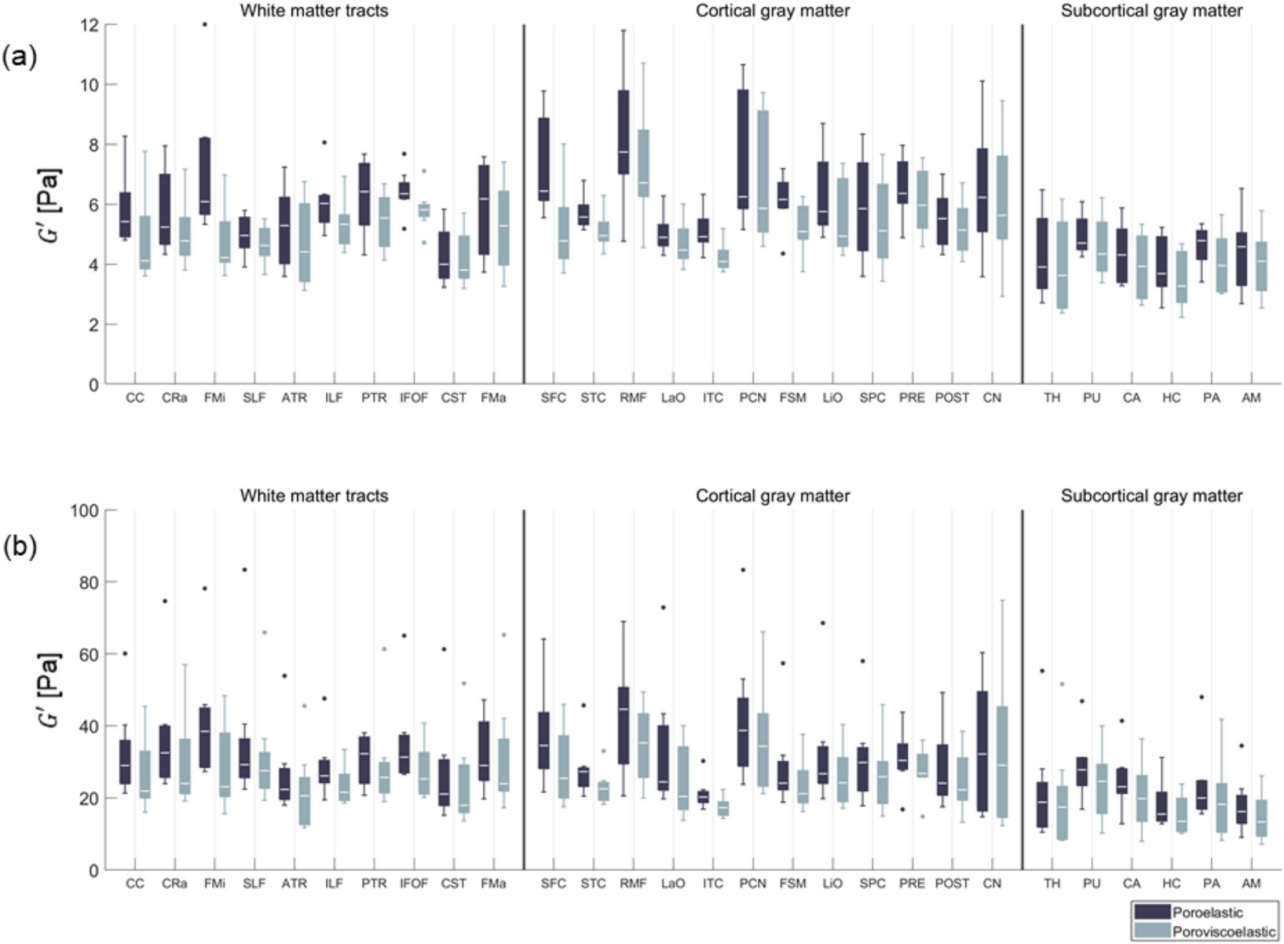
Boxplots illustrating the mean regional storage modulus across minor regions for all subjects for the 1‐Hz motion component in (a) and 2‐Hz motion component in (b). The white line inside the boxplots represents the median value, while the upper and lower ends of the boxplots correspond to the 25th and 75th quartiles. The whiskers represent the maximum and minimum values which are not outliers (presented as dots and are defined as values that lie more than 1.5·IQR away from the upper or lower quartiles).

All regions were successfully identified within the property maps for each scan. Figure 4a presents boxplots illustrating the material property values across major regions for all subjects for the 1‐Hz motion component, while Figure 4b shows the corresponding boxplots for the 2‐Hz motion component. Table S1 contains the corresponding mean and standard deviation for each boxplot. Similar regional trends were observed for the majority of the properties for both motion components. The poroviscoelastic model generally yielded lower storage modulus and hydraulic permeability values compared to the poroelastic model, though it produced slightly higher lambda modulus values. Large differences in storage modulus were observed between the two motion components, with the 2 Hz component demonstrating approximately five times higher values for both models. Boxplots displaying the storage modulus values across all minor regions for all subjects are presented in Figure 5 for both motion components. Similar regional trends were observed across both material models and motion components, with the CGM generally being the stiffest region and the SGM the softest. In accordance with what was found for the major regions, the poroviscoelastic model consistently yielded lower storage modulus values for all minor regions. The mean and standard deviation across material properties for all brain regions are presented in Tables S2–S4.

### 3.4 Repeatability Analysis

**FIGURE 6:**
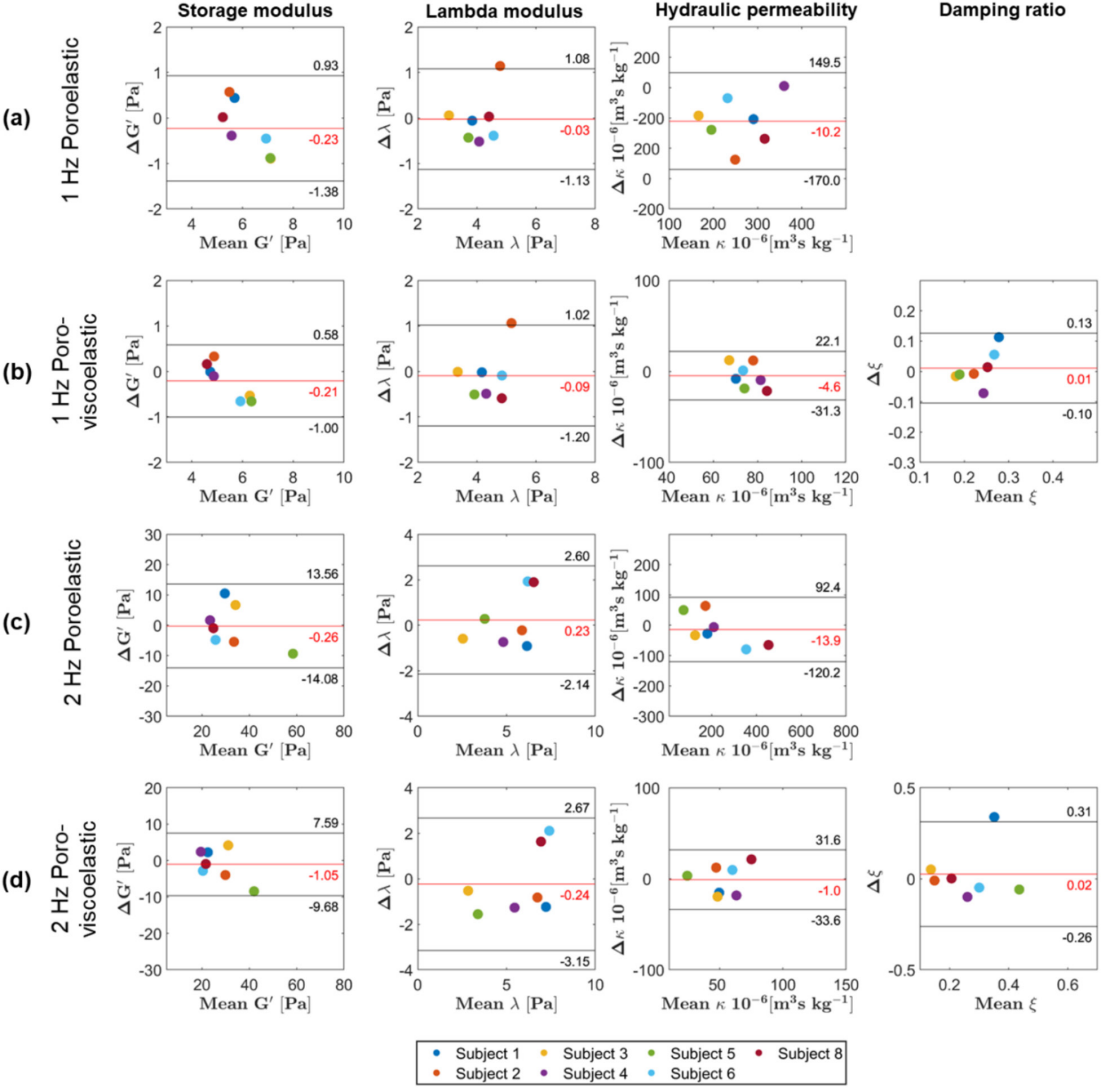
Subject‐wise Bland–Altman plots for the whole‐brain mean material properties for 1‐Hz poroelastic reconstruction (a), 1‐Hz poroviscoelastic reconstruction (b), 2‐Hz poroelastic reconstruction (c), and 2‐Hz poroviscoelastic reconstruction (d). The horizontal red line demonstrates the bias b which is the mean of the differences between two repeated measurements, and the upper and lower horizontal black lines illustrate the upper and lower limits of agreement respectively, defined as b±1.96·std.

Figure 6 presents subject‐wise Bland–Altman plots for the whole‐brain mean material properties, spanning both material models and driving frequencies. Black horizontal lines indicate the limits of agreement in each plot. These limits are further detailed in Table 2 for the 1‐Hz motion component and Table 3 for the 2‐Hz motion component, both expressed relative to the mean whole‐brain values. Additionally, the limits of agreements across major regions are also included in both tables.

**TABLE 2:** Table containing the limits of agreement (upper/lower) for all material properties across the major regions for the 1‐Hz motion component, expressed relative to the mean whole‐brain values.

	Region	Poroelastic	Poroviscoelastic
Storage modulus	Global	0.151/−0.225	0.109/−0.187
	SGM	0.173/−0.262	0.190/−0.327
	CGM	0.236/−0.292	0.157/−0.209
	WMT	0.138/−0.200	0.125/−0.242
Lambda modulus	Global	0.266/−0.279	0.233/−0.275
	SGM	0.454/−0.410	0.313/−0.293
	CGM	0.277/−0.285	0.252/−0.279
	WMT	0.238/−0.326	0.157/‐0.243
Hydraulic permeability	Global	0.578/−0.657	0.292/−0.415
	SGM	1.612/−1.545	0.496/−0.930
	CGM	0.532/−0.710	0.723/−0.895
	WMT	0.992/−0.754	0.204/−0.181
Damping ratio	Global	N/A	0.541/−0.447
	SGM	N/A	0.805/−0.579
	CGM	N/A	0.707/−0.710
	WMT	N/A	0.821/−0.473

**TABLE 3:** Table containing the limits of agreement (upper/lower) for all material properties across the major regions for the 2‐Hz motion component, expressed relative to the mean whole‐brain values.

	Region	Poroelastic	Poroviscoelastic
Storage modulus	Global	0.413/−0.429	0.284/−0.362
	SGM	0.690/−0.785	0.711/−0.864
	CGM	0.514/−0.557	0.406/−0.526
	WMT	0.579/−0.580	0.496/−0.723
Lambda modulus	Global	0.508/−0.417	0.467/−0.551
	SGM	0.506/−0.513	0.498/−0.927
	CGM	0.815/−0.560	0.653/−0.574
	WMT	0.493/−0.387	0.465/−0.542
Hydraulic permeability	Global	0.411/−0.535	0.602/−0.638
	SGM	1.423/−1.526	0.631/−1.086
	CGM	0.956/−0.812	0.709/−0.894
	WMT	1.219/−1.030	0.977/−0.571
Damping ratio	Global	N/A	1.189/−1.000
	SGM	N/A	3.371/−2.376
	CGM	N/A	0.841/−0.853
	WMT	N/A	1.898/−1.190

### 3.5 Symmetry Analysis

**FIGURE 7:**
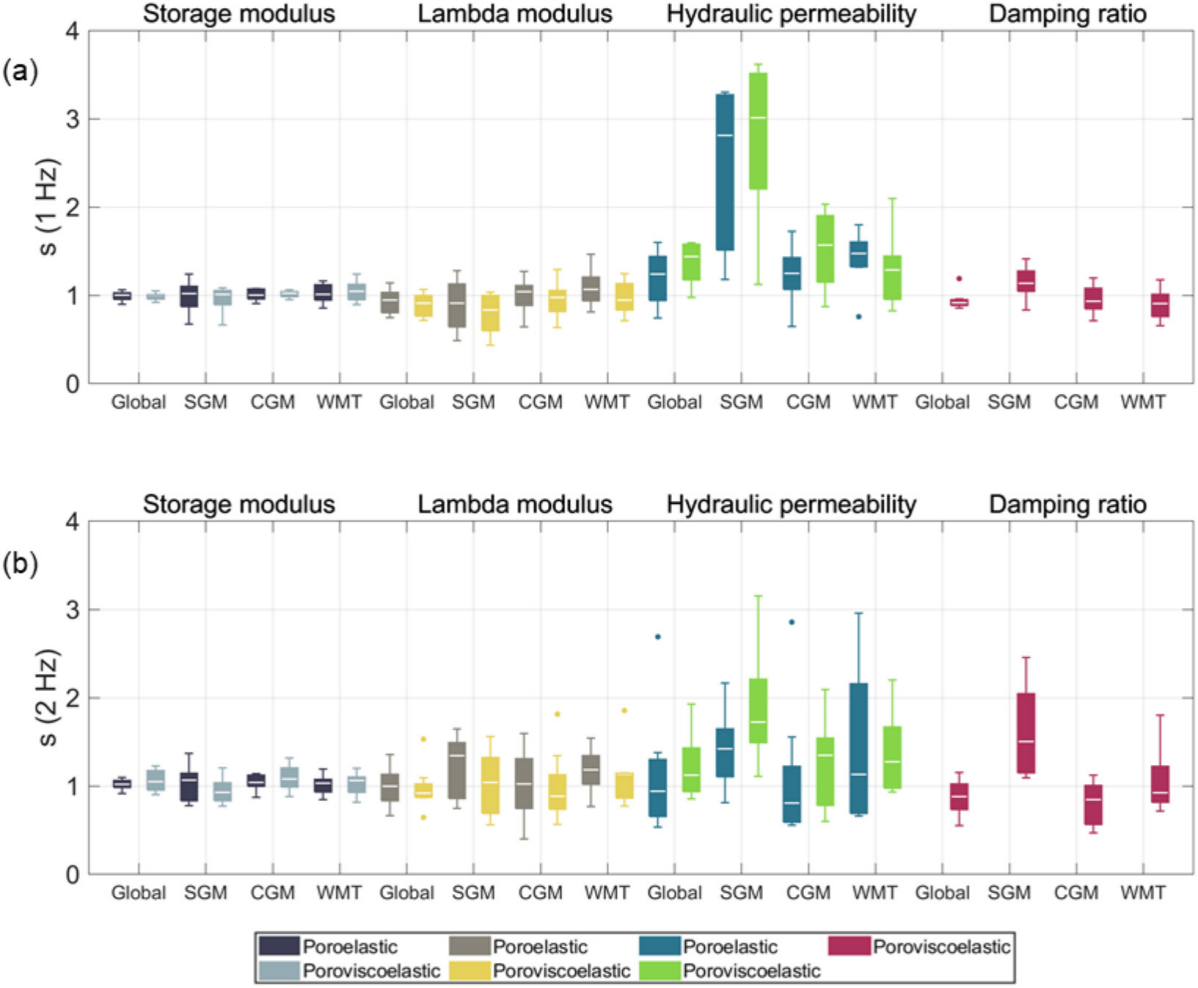
Boxplots displaying the mean regional symmetry ratio distributions across major regions for all subjects for the 1‐Hz motion component (top) and 2‐Hz motion component (bottom). Higher symmetry ratios correspond to the right‐hand side of the brain containing higher values of the material property. The white line inside the boxplots represents the median value, while the upper and lower ends of the boxplots correspond to the 25th and 75th quartiles. The whiskers represent the maximum and minimum values which are not outliers (presented as dots and are defined as values that lie more than 1.5·IQR away from the upper or lower quartiles).

The symmetry ratio was computed for all material properties and is presented in Figure 7 as boxplots, showing the symmetry ratio distribution across all subjects for different major regions, material models, and motion components. Tables S5 and S6 contain the corresponding mean and standard deviation for each boxplot. Overall, the storage modulus demonstrated good symmetry in both CGM and WMT, with median values near zero and minimal intersubject variability. In contrast, greater asymmetry was observed in the SGM, with shifted medians and higher intersubject variability, a trend consistently seen across all other properties. The lambda and loss moduli demonstrated less symmetry than the storage modulus in all regions, and the hydraulic permeability exhibited the highest asymmetry of all material properties. The storage modulus displayed similar symmetry ratios across models, though the poroviscoelastic model yielded slightly better symmetry for both motion components. For the lambda modulus and hydraulic permeability, the poroviscoelastic model yielded generally less symmetry for both motion components.

### 3.6 Influence of Hydraulic Permeability

**FIGURE 8:**
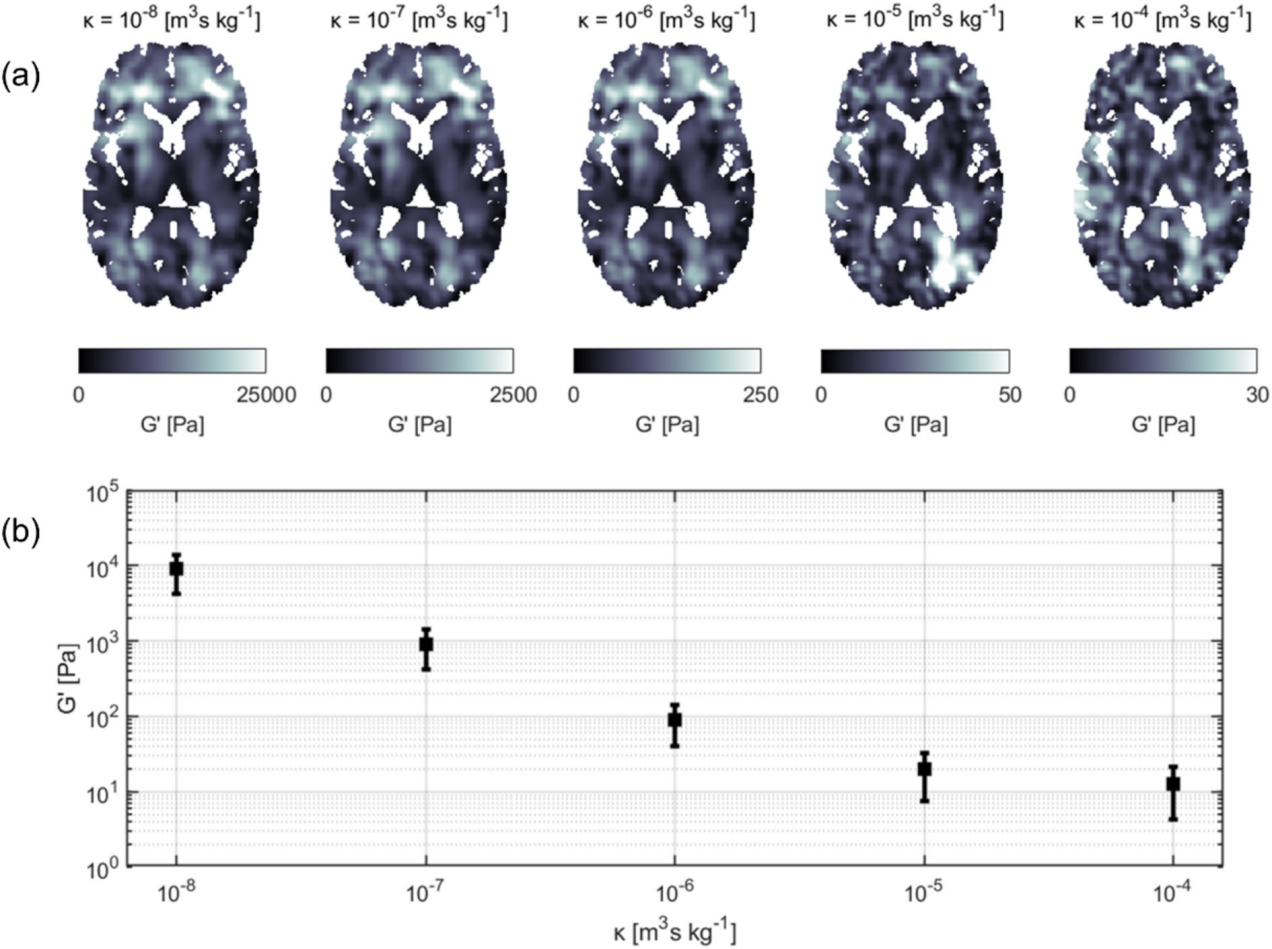
Representative axial slices of storage modulus maps for varying mean global values of hydraulic permeability in a single subject (a). The corresponding global mean storage modulus are shown in (b), where the whiskers represent the standard deviation.

Figure 8a displays representative axial slices of storage modulus maps for varying mean global values of hydraulic permeability in a single subject. The maps show increasing spatial heterogeneity as the hydraulic permeability is increased, particularly for $\kappa =10^{-5}$ and $\kappa =10^{-4}$ m^3^s kg^−1^. Figure 8b shows the corresponding global mean storage modulus, where the whiskers represent the standard deviation. The storage modulus demonstrates high sensitivity to varying levels of hydraulic permeability.

## 4 Discussion

This study evaluated the performance and reliability of poroelastic and poroviscoelastic brain models for 7 T iMRE with test–retest repeatability analysis, while also assessing how the reconstructed material properties are affected by different driving frequencies and noise levels. The material properties were successfully reconstructed for both brain models and driving frequencies for all subjects. The results demonstrated good consistency in material properties across repeated scan sessions, as evidenced by the relatively low LoA particularly for the storage modulus. Additionally, the storage modulus exhibited good left–right symmetry across models and driving frequencies, while other properties showed greater asymmetry. The poroelastic and poroviscoelastic models produced generally similar results, with the largest differences observed in hydraulic permeability. On average, the poroviscoelastic model yielded lower values for the storage modulus and hydraulic permeability across all regions, while slightly higher values were observed for the lambda modulus. It also demonstrated slightly better repeatability across material properties compared to the poroelastic model, though symmetry was comparable or slightly worse. The material properties derived from the 2‐Hz motion component showed higher storage modulus and lower hydraulic permeability, consistent with the frequency‐related stiffening associated with porous media [31]. Their spatial distributions were similar to those from the 1‐Hz component across both models, albeit at reduced repeatability and symmetry. This suggests that a significant portion of the cardiac signal is still captured in the 2‐Hz motion component, which highlights its potential for use in multifrequency reconstruction [32].

In contrast to previous attempts at iMRE using a viscoelastic model [14], the porous models recover unique solutions with consistent property convergence across all subjects. This is because the elastic forces can be balanced by fluid–solid interaction forces even at low actuation frequencies, a mechanism absent in the viscoelastic model. An interesting consequence is that this may shed some more light on the stiffness of the brain within its natural environment, as brain stiffness is characterized at a physiologically relevant frequency. This contrasts with extrinsic MRE, which typically recovers brain stiffness on the order of kPa [27, 33, 34] due to the complex shear modulus increasing strongly with actuation frequency [31]. Furthermore, previous studies on poroelastic iMRE fixed a global value for the hydraulic permeability of $\kappa =10^{-7}$ m^3^ s kg^−1^ rather than performing a voxel‐wise reconstruction during the inversion process (M. [18]). This approach resulted in higher recovered stiffness values, within the kilopascal range, which scaled with the chosen hydraulic permeability. A similar relationship was observed between stiffness and the assigned hydraulic permeability for our data, as highlighted in Figure 8. Instead, by allowing hydraulic permeability to be determined with the other mechanical properties, we find very low values for the complex shear modulus, with a mean storage modulus of around 5–6 Pa for the whole brain at an estimated hydraulic permeability in the order of 10^−4^ m^3^ s kg^−1^. Although very low, these values show some similarity with values reported by Herthum et al., who reported ultra‐soft stiffness obtained by performing a wavelength analysis in intrinsic activation using steady‐state MRE [31]. Using viscoelastic model‐fitting, they found a shear modulus of 42 ± 13 Pa. While this is higher than what we report, they noted that they may have overestimated their values due to misplacement of the image plane. Furthermore, we reconstructed the complex shear modulus of the drained porous solid rather than the saturated matrix, which could also play a role in the observed differences. In both cases, the measured stiffness points towards the brain exhibiting ultra‐soft properties when unperturbed by external vibrations with relatively high frequencies.

For both porous models, the CGM region exhibited higher storage modulus values than the SGM region. This contrasts with what was found in viscoelastic reconstruction, where the SGM region showed higher stiffnesses. Despite this discrepancy, the relative storage modulus across SGM subregions with respect to each other aligns well with findings from both intrinsic and standard MRE [14, 27]. The agreement between porous and viscoelastic models is generally less consistent across CGM regions, likely due to differences in how the models interpret the complex geometry of gyri and sulci in the CGM. Thus, while the relative stiffness of subregions within the SGM and CGM shows good agreement with viscoelastic reconstruction, the overall stiffness across the entire SGM and CGM regions differs between porous and viscoelastic models. This suggests that these discrepancies may arise from differences in fluid motion dynamics between tissue types and how the different material models treat these fluid–solid interactions differently. Using the porous models, the shear modulus is reconstructed for the drained porous solid, while for the viscoelastic model, the storage modulus is reconstructed for the fully saturated matrix. Consequently, some amount of discrepancy between poroelastic and viscoelastic reconstruction is expected.

Given the visual similarity in the spatial patterns of the damping ratio maps in the poroviscoelastic model and the hydraulic permeability maps in the poroelastic model, it seems that a portion of the effective mechanical attenuation depicted in the hydraulic permeability maps of the poroelastic model is accounted for by increased damping in the poroviscoelastic model. This effect is clearly expressed in the anterior region of the brain and surrounding the ventricles (Figure 3), where there are high‐valued regions along with considerable intersubject variability, causing artifact‐like structures in the mean maps for the poroelastic model. In the poroviscoelastic model, where the damping ratio expresses part of the mechanical attenuation, such artifacts disappear and the intersubject variability decreases. Subsequently, regions with very high hydraulic permeability for the poroelastic model instead become apparent in the mean damping ratio maps for the poroviscoelastic model. This is particularly the case surrounding the frontal lobe, where the damping ratio reaches exceedingly high values in the anterior side of the ventricles, and on either side of the hemispheric fissure. This region of high damping ratio is also observed using a viscoelastic model in iMRE [14] and likely arises for two main reasons. First, in and around the corpus callosum, high damping ratio is likely exhibited due to destructive interference between the displacement waves propagating inwards from the right‐ and left‐hand sides of the brain. Secondly, as the Falx Cerebri is very stiff in comparison to the surrounding brain tissue, right–left motions in this region rapidly decrease to zero, which gets interpreted as a viscous effect during property reconstruction. These effects are much less prominent in the posterior region of the brain, where right–left motion amplitudes are relatively smaller.

The storage modulus demonstrates relatively good repeatability for the poroviscoelastic model, as indicated by relatively low whole‐brain LoA (upper/lower): 10.9%/−18.5% and slightly worse repeatability for the poroelastic model with a whole‐brain LoA of 15.7% /22.2%, both relative to the mean whole‐brain storage modulus. In comparison, a recent study on the repeatability of extrinsic MRE using a viscoelastic model reported whole‐brain LoA of 9.6%/6.2% relative to the whole‐brain mean for the storage modulus, using a 3‐T scanner and a 50‐Hz actuation frequency in 15 healthy subjects [35]. The hydraulic permeability showed the worst repeatability out of all properties for the poroelastic model but was substantially more repeatable using the poroviscoelastic model. This is likely because in the poroelastic model, the hydraulic permeability seems to account for the mechanical attenuation effects, whereas in the poroviscoelastic model, these attenuation effects are separated into distinct viscoelastic and poroelastic components, allowing the hydraulic permeability to primarily reflect fluid motion rather than the combined influence of both fluid movement and tissue viscosity. This separation reduces the complexity and variability of the hydraulic permeability measurements, improving repeatability. The whole‐brain damping ratio using the poroviscoelastic model demonstrates comparatively poor repeatability (LoA: 54.0%/−44.7%), comparable to what was found using a viscoelastic model (LoA: 51.2%/−47.0%) [14]. This issue arises partly because the damping ratio is derived from two reconstructed parameters, the storage and loss moduli, causing noise to propagate from both sources. In addition, the low wave speed of cerebral arterial pulsations should be considered, which are substantially lower than the mechanical waves applied in standard MRE. Since the loss modulus is linked to the velocity of the propagating wave, extremely low velocities introduce greater uncertainty in the loss modulus measurements, leading to higher inconsistency in the damping ratio. This is a fundamental limitation of iMRE and explains in part the interest in poroelastic material models at these low frequencies ([14]; M. [18, 20, 26]).

The symmetry analysis revealed patterns consistent with those observed in the repeatability analysis. Among the material properties, the storage modulus exhibited the highest level of symmetry, and the hydraulic permeability showed the least symmetry. This is likely related to differences in the relative timing between the right and left hemispheres when the cardiac‐induced arterial pulse enters the brain. Because the heart is situated on the left‐hand side of the body, the distance from the aortic arch to the skull base is slightly larger for the right side than for the left (22.2 ± 2.2 and 20.8 ± 1.9 cm, respectively) [36], resulting in a delay in the pulse pressure wave in the right hemisphere. Furthermore, there is also a difference in early systolic pulse wave velocity between the right and left carotid arteries of 5.29 and 5.63 m/s, respectively (L.‐X. [37]), which further adds to the asymmetry. For this reason, some degree of hemispheric asymmetry may be expected in the fluid‐related properties. Still, the pronounced asymmetry observed in the hydraulic permeability and lambda modulus is likely amplified by the pressure boundary conditions, as both properties are related to fluid motion. In the current implementation, as the pressure on the boundaries is unknown, they are assumed to be 0 across all subzones, thus assuming symmetrical pressures despite asymmetrical motions. This simplification overlooks the physiological differences in local pressure and potentially leads to inaccuracies in the reconstructed properties related to fluid flow, such as the hydraulic permeability and the lambda modulus. Alternative pressure boundary condition formulations have been proposed in the past by Tan et al. [26]; however, this approach, based on the direct use of differentiated data within the inversion formulation, led to instabilities when treating the data presented here. Recent development of coupled adjoint‐field formulations for the subzone gradient‐based inversion scheme removes the requirement for boundary conditions on the displacement field in the gradient calculation for nonlinear inversion [38]. Similar formulations for the governing poroelastic equations, coupled with the pressure‐forward problem described by Tan et al. in 2017, would similarly remove the requirement for boundary conditions on the pressure field for the poroelastic system and provide a robust approach to the reconstruction of physiologically relevant internal pressure measurements via MRE. This may prove to be a key step in reconstructing robust and accurate material property maps using poroelastic models.

The property maps recovered from the 2‐Hz motion component generally exhibited spatial distributions similar to those observed with the 1 Hz component, though with reduced detail. This difference was particularly evident in the regional analysis, where the relative mean storage modulus across major regions appears more homogeneous compared to the 1‐Hz case. At the same time, very similar regional trends were recovered across motion components, both in the major regions across all material properties and for the minor regions for the storage modulus. Remarkably, the storage modulus recovered using the 2‐Hz component exhibited approximately five times higher values than for the 1‐Hz component for both material models. This is consistent with the frequency‐related stiffening that is associated with porous media, as reported by Herthum et al. [31]. Although the underlying mechanism probably is multifactorial, a large contributing factor is likely the impact of higher actuation frequencies on fluid‐matrix interactions. At higher frequencies, the reduced time available for fluid redistribution within the porous network leads to greater confinement of the fluid within the solid matrix. This confinement enhances the coupling between the fluid and solid phases, resulting in increased effective stiffness and decreased hydraulic permeability. These effects are consistent with our findings, where the hydraulic permeability is lower for the 2‐Hz motion component in both material models. Notably, the lambda modulus of the solid matrix does not change significantly with frequency, which may be due to the fluid–solid coupling effect being primarily felt in the shear resistance or due to the poorer quality of the divergence field measurements compared to the strain deformation field. Overall, although the 2‐Hz component is expected to generate considerably noisier property maps than the 1‐Hz component, many of the prominent features are preserved across motion components. Therefore, as much of the information is preserved in the 2‐Hz motion component, it is likely beneficial to include this frequency component for future implementation of multifrequency reconstruction [32].

Both the poroelastic model and the poroviscoelastic model exhibited good performance on several fronts. Two of the main limitations encountered using a viscoelastic model were resolved, namely the problem of nonunique solutions and the existence of high‐valued artifact‐like *hotspots* in the stiffness maps [14]. Overall, the poroviscoelastic model outperformed the poroelastic model in the current implementation in terms of consistency of spatial distribution, repeatability, and symmetry of the storage modulus. Thus, despite being more complex, the poroviscoelastic model seemingly exhibits more consistency while also providing added details about the mechanical properties of the brain. Similar conclusions were reached by Greiner et al. [39], who found that a poroviscoelastic model better captured time‐dependent material behavior compared to a purely poroelastic model. At the same time, both models exhibited limitations in their current implementation. More specifically, the assumption of having the pressure field boundary conditions as p=0 has large effects during nonlinear inversion, affecting the reconstructed material property maps with the flow related properties being the most influenced. The effects of this assumption are likely reflected in the quality of the lambda modulus and hydraulic permeability maps, where the repeatability and symmetry were considerably worse than for the storage modulus. Therefore, the natural step forward is to, as previously discussed, develop a coupled adjoint‐field formulation [38] for the nonlinear inversion scheme in combination with the pressure‐forward problem [26]. This approach would remove the necessity for pressure boundary conditions for both the poroelastic and poroviscoelastic models and rectify certain inconsistencies encountered in this work. Furthermore, the results presented here have not been validated using phantom or simulation studies, which would be essential for further assessing the accuracy and robustness of both models and are to be addressed in future studies.

## 5 Conclusion

In this work, the mechanical properties of the brain were successfully reconstructed using poroelastic and poroviscoelastic models and iMRE at 7 T. Notably, this is the first application of a poroviscoelastic model in brain MRE, in addition to the first time that the hydraulic permeability has been reconstructed on a voxel‐wise basis. The performance and reliability of the poroelastic and poroviscoelastic models were compared, with the poroviscoelastic model generally found to be more suitable for iMRE in the current implementation. Additionally, the use of the 2‐Hz motion component in the nonlinear inversion algorithm was explored and found to provide reasonable mechanical property maps, suggesting that it could be advantageous for future multifrequency reconstruction approaches. The main limitation of this work includes the assumption on the boundary conditions of the pressure field. Future work thus aims to develop a coupled adjoint‐field formulation in combination with the pressure‐forward problem for the nonlinear inversion scheme, as well as investigating multifrequency inversion approaches.

## Ethics Statement

The imaging performed for this study was done under the research proposal “MRI protocol development for all field strengths”, approved by the local Ethics Review Board of the UMC Utrecht (METC 15‐466).

## Consent

All subjects gave written informed consent for participation in this study, which was approved by the ethical review board of UMC Utrecht.

## Conflicts of Interest

The authors declare no conflicts of interest.

## Permission to Reproduce Material From Other Sources

N/A.

## Supporting information


**Figure S1** Representative axial slices of the storage modulus (a), lambda modulus (b), hydraulic permeability (c), and damping ratio (d) obtained from the 2‐Hz motion component for one subject along with a repeated scan which has been co‐registered to the first scan. The equivalent slice of the T1‐weighted image is shown in (f) for anatomical reference.
**Table S1:** The mean material properties and corresponding standard deviation across major brain regions for both material models and motion frequency components.
**Table S2:** The mean material properties and corresponding standard deviation across brain regions in subcortical gray matter for both material models and motion frequency components.
**Table S3:** The mean material properties and corresponding standard deviation across brain regions in cortical gray matter for both material models and motion frequency components.
**Table S4:** The mean material properties and corresponding standard deviation across brain regions in white matter tracts for both material models and motion frequency components.
**Table S5:** The mean and standard deviation of the symmetry ratio for all material properties across the major regions for the 1‐Hz motion component.
**Table S6:** The mean and standard deviation of the symmetry ratio for all material properties across the major regions for the 2‐Hz motion component.

## Data Availability

The data presented in this study are available from the corresponding author upon request.

## References

[1] K. J. Glaser , A. Manduca , and R. L. Ehman , “Review of MR Elastography Applications and Recent Developments,” Journal of Magnetic Resonance Imaging : JMRI 36, no. 4 (2012): 757–774, 10.1002/jmri.23597. 22987755 PMC3462370

[2] L. V. Hiscox , C. L. Johnson , E. Barnhill , et al., “Magnetic Resonance Elastography (MRE) of the Human Brain: Technique, Findings and Clinical Applications,” Physics in Medicine and Biology 61, no. 24 (2016): R401–R437, 10.1088/0031-9155/61/24/R401. 27845941

[3] O. Rouvière , M. Yin , M. A. Dresner , et al., “MR Elastography of the Liver: Preliminary Results,” Radiology 240, no. 2 (2006): 440–448, 10.1148/radiol.2402050606. 16864671

[4] M. Yin , J. Woollard , X. Wang , et al., “Quantitative Assessment of Hepatic Fibrosis in an Animal Model With Magnetic Resonance Elastography,” Magnetic Resonance in Medicine 58, no. 2 (2007): 346–353, 10.1002/mrm.21286. 17654577

[5] A. Bunevicius , K. Schregel , R. Sinkus , A. Golby , and S. Patz , “REVIEW: MR Elastography of Brain Tumors,” NeuroImage: Clinical 25 (2020): 102109, 10.1016/j.nicl.2019.102109. 31809993 PMC6909210

[6] Y. Feng , M. C. Murphy , E. Hojo , F. Li , and N. Roberts , “Magnetic Resonance Elastography in the Study of Neurodegenerative Diseases,” Journal of Magnetic Resonance Imaging : JMRI 59, no. 1 (2024): 82–96, 10.1002/jmri.28747. 37084171

[7] N. S. Farhat , R. Theiss , T. Santini , T. S. Ibrahim , and H. J. Aizenstein , “Neuroimaging of Small Vessel Disease in Late‐Life Depression,” Advances in Experimental Medicine and Biology 1192 (2019): 95–115, 10.1007/978-981-32-9721-0_5. 31705491 PMC6939470

[8] J. M. Barnes , L. Przybyla , and V. M. Weaver , “Tissue Mechanics Regulate Brain Development, Homeostasis and Disease,” Journal of Cell Science 130, no. 1 (2017): 71–82, 10.1242/jcs.191742. 28043968 PMC5394781

[9] R. Forouhandehpour , M. Bernier , G. Gilbert , R. Butler , K. Whittingstall , and E. Van Houten , “Cerebral Stiffness Changes During Visual Stimulation: Differential Physiological Mechanisms Characterized by Opposing Mechanical Effects,” Neuroimage: Reports 1, no. 2 (2021): 100014, 10.1016/j.ynirp.2021.100014. PMC12172913 40567862

[10] A. Goriely , M. G. D. Geers , G. A. Holzapfel , et al., “Mechanics of the Brain: Perspectives, Challenges, and Opportunities,” Biomechanics and Modeling in Mechanobiology 14, no. 5 (2015): 931–965, 10.1007/s10237-015-0662-4. 25716305 PMC4562999

[11] P. S. Lan , K. J. Glaser , R. L. Ehman , and G. H. Glover , “Imaging Brain Function With Simultaneous BOLD and Viscoelasticity Contrast: fMRI/fMRE,” NeuroImage 211 (2020): 116592, 10.1016/j.neuroimage.2020.116592. 32014553 PMC7153752

[12] H. Schwarb , C. L. Johnson , M. D. J. McGarry , and N. J. Cohen , “Medial Temporal Lobe Viscoelasticity and Relational Memory Performance,” NeuroImage 132 (2016): 534–541, 10.1016/j.neuroimage.2016.02.059. 26931816 PMC4970644

[13] T. Meyer , J. Castelein , J. Schattenfroh , et al., “Magnetic Resonance Elastography in a Nutshell: Tomographic Imaging of Soft Tissue Viscoelasticity for Detecting and Staging Disease With a Focus on Inflammation,” Progress in Nuclear Magnetic Resonance Spectroscopy 144‐145 (2024): 1–14, 10.1016/j.pnmrs.2024.05.002. 39645347

[14] M. Burman Ingeberg , E. Van Houten , and J. J. M. Zwanenburg , “Estimating the Viscoelastic Properties of the Human Brain at 7 T MRI Using Intrinsic MRE and Nonlinear Inversion,” Human Brain Mapping 44, no. 18 (2023): 6575–6591, 10.1002/hbm.26524. 37909395 PMC10681656

[15] L. M. Solamen , M. D. J. McGarry , J. Fried , J. B. Weaver , S. S. Lollis , and K. D. Paulsen , “Poroelastic Mechanical Properties of the Brain Tissue of Normal Pressure Hydrocephalus Patients During Lumbar Drain Treatment Using Intrinsic Actuation MR Elastography,” Academic Radiology 28, no. 4 (2021): 457–466, 10.1016/j.acra.2020.03.009. 32331966 PMC7575616

[16] J. B. Weaver , A. J. Pattison , M. D. McGarry , et al., “Brain Mechanical Property Measurement Using MRE With Intrinsic Activation,” Physics in Medicine and Biology 57, no. 22 (2012): 7275–7287, 10.1088/0031-9155/57/22/7275. 23079508 PMC3797022

[17] A. Zorgani , R. Souchon , A.‐H. Dinh , et al., “Brain Palpation From Physiological Vibrations Using MRI,” Proceedings of the National Academy of Sciences of the United States of America 112, no. 42 (2015): 12917–12921, 10.1073/pnas.1509895112. 26438877 PMC4620887

[18] M. McGarry , E. Van Houten , L. Solamen , S. Gordon‐Wylie , J. Weaver , and K. Paulsen , “Uniqueness of Poroelastic and Viscoelastic Nonlinear Inversion MR Elastography at Low Frequencies,” Physics in Medicine and Biology 64, no. 7 (2019): 075006, 10.1088/1361-6560/ab0a7d. 30808018

[19] M. D. J. McGarry , C. L. Johnson , B. P. Sutton , et al., “Suitability of Poroelastic and Viscoelastic Mechanical Models for High and Low Frequency MR Elastography,” Medical Physics 42, no. 2 (2015): 947–957, 10.1118/1.4905048. 25652507 PMC4312344

[20] D. R. Sowinski , M. D. J. McGarry , E. E. W. Van Houten , S. Gordon‐Wylie , J. B. Weaver , and K. D. Paulsen , “Poroelasticity as a Model of Soft Tissue Structure: Hydraulic Permeability Reconstruction for Magnetic Resonance Elastography In Silico,” Frontiers in Physics 8 (2021): 617582, 10.3389/fphy.2020.617582. 36340954 PMC9635531

[21] A. L. Adams , M. A. Viergever , P. R. Luijten , and J. J. M. Zwanenburg , “Validating Faster DENSE Measurements of Cardiac‐Induced Brain Tissue Expansion as a Potential Tool for Investigating Cerebral Microvascular Pulsations,” NeuroImage 208 (2020): 116466, 10.1016/j.neuroimage.2019.116466. 31843712

[22] J. D'Errico , “Inpainting NaN Elements in 3‐D,” (2024), MATLAB Central File Exchange. (Retrieved November 27, 2024), https://www.mathworks.com/matlabcentral/fileexchange/21214‐inpainting‐nan‐elements‐in‐3‐d.

[23] M. D. J. McGarry , E. E. W. Van Houten , C. L. Johnson , et al., “Multiresolution MR Elastography Using Nonlinear Inversion,” Medical Physics 39, no. 10 (2012): 6388–6396, 10.1118/1.4754649. 23039674 PMC3477197

[24] E. E. Van Houten , M. I. Miga , J. B. Weaver , F. E. Kennedy , and K. D. Paulsen , “Three‐Dimensional Subzone‐Based Reconstruction Algorithm for MR Elastography,” Magnetic Resonance in Medicine 45, no. 5 (2001): 827–837, 10.1002/mrm.1111. 11323809

[25] P. R. Perriñez , F. E. Kennedy , E. E. W. Van Houten , J. B. Weaver , and K. D. Paulsen , “Magnetic Resonance Poroelastography: An Algorithm for Estimating the Mechanical Properties of Fluid‐Saturated Soft Tissues,” IEEE Transactions on Medical Imaging 29, no. 3 (2010): 746–755, 10.1109/TMI.2009.2035309. 20199912 PMC2865251

[26] L. Tan , M. D. J. McGarry , E. E. W. Van Houten , et al., “A Numerical Framework for Interstitial Fluid Pressure Imaging in Poroelastic MRE,” PLoS ONE 12, no. 6 (2017): e0178521, 10.1371/journal.pone.0178521. 28586393 PMC5460821

[27] L. V. Hiscox , M. D. J. McGarry , H. Schwarb , et al., “Standard‐Space Atlas of the Viscoelastic Properties of the Human Brain,” Human Brain Mapping 41, no. 18 (2020): 5282–5300, 10.1002/hbm.25192. 32931076 PMC7670638

[28] A. L. Manera , M. Dadar , V. Fonov , and D. L. Collins , “CerebrA, Registration and Manual Label Correction of Mindboggle‐101 Atlas for MNI‐ICBM152 Template,” Scientific Data 7, no. 1 (2020): 237, 10.1038/s41597-020-0557-9. 32669554 PMC7363886

[29] S. Mori , K. Oishi , H. Jiang , et al., “Stereotaxic White Matter Atlas Based on Diffusion Tensor Imaging in an ICBM Template,” NeuroImage 40, no. 2 (2008): 570–582, 10.1016/j.neuroimage.2007.12.035. 18255316 PMC2478641

[30] S. Mori , S. Wakana , L. M. Nagae‐Poetscher , and P. C. M. van Zijl , “MRI Atlas of Human White Matter,” American Journal of Neuroradiology 27, no. 6 (2006): 1384–1385.

[31] H. Herthum , S. C. H. Dempsey , A. Samani , et al., “Superviscous Properties of the In Vivo Brain at Large Scales,” Acta Biomaterialia 121 (2021): 393–404, 10.1016/j.actbio.2020.12.027. 33326885

[32] S. Papazoglou , S. Hirsch , J. Braun , and I. Sack , “Multifrequency Inversion in Magnetic Resonance Elastography,” Physics in Medicine and Biology 57, no. 8 (2012): 2329–2346, 10.1088/0031-9155/57/8/2329. 22460134

[33] C. L. Johnson , M. D. J. McGarry , E. E. W. Van Houten , et al., “Magnetic Resonance Elastography of the Brain Using Multishot Spiral Readouts With Self‐Navigated Motion Correction,” Magnetic Resonance in Medicine 70, no. 2 (2013): 404–412, 10.1002/mrm.24473. 23001771 PMC3529970

[34] J. Zhang , M. A. Green , R. Sinkus , and L. E. Bilston , “Viscoelastic Properties of Human Cerebellum Using Magnetic Resonance Elastography,” Journal of Biomechanics 44, no. 10 (2011): 1909–1913, 10.1016/j.jbiomech.2011.04.034. 21565346

[35] S. F. Svensson , J. De Arcos , O. I. Darwish , et al., “Robustness of MR Elastography in the Healthy Brain: Repeatability, Reliability, and Effect of Different Reconstruction Methods,” Journal of Magnetic Resonance Imaging : JMRI 53, no. 5 (2021): 1510–1521, 10.1002/jmri.27475. 33403750

[36] F. A. Choudhry , J. T. Grantham , A. T. Rai , and J. P. Hogg , “Vascular Geometry of the Extracranial Carotid Arteries: An Analysis of Length, Diameter, and Tortuosity,” Journal of Neurointerventional Surgery 8, no. 5 (2016): 536–540, 10.1136/neurintsurg-2015-011671. 25841169

[37] L.‐X. Yin , C.‐Y. Ma , S. Wang , et al., “Reference Values of Carotid Ultrafast Pulse‐Wave Velocity: A Prospective, Multicenter, Population‐Based Study,” Journal of the American Society of Echocardiography 34, no. 6 (2021): 629–641, 10.1016/j.echo.2021.01.003. 33422666

[38] S. Kurtz , B. Wattrisse , and E. E. W. Van Houten , “Minimizing Measurement‐Induced Errors in Viscoelastic MR Elastography,” IEEE Transactions on Medical Imaging 43, no. 3 (2024): 1138–1148, 10.1109/TMI.2023.3329293. 37910409

[39] A. Greiner , N. Reiter , J. Hinrichsen , et al., “Model‐Driven Exploration of Poro‐Viscoelasticity in Human Brain Tissue: Be Careful With the Parameters!,” Interface Focus 14, no. 6 (2024): 20240026, 10.1098/rsfs.2024.0026. 39649453 PMC11620825

