# Supplementary material for: Evaluating the Performance and Repeatability of Poroelastic and Poroviscoelastic Models in Intrinsic MR Elastography
Source: NMR Biomed. 2025 Jun 6;38(7):e70073. doi: 10.1002/nbm.70073 (PMC12142453; doi:10.1002/nbm.70073)
Supplement: Supplementary file 1 — Figure S1 Representative axial slices of the storage modulus (a), lambda modulus (b), hydraulic permeability (c), and damping ratio (d) obtained from the 2‐Hz motion component for one subject along with a repeated scan which has been co‐registered to the first scan. The equivalent slice of the T1‐weighted image is shown in (f) for anatomical reference. Table S1: The mean material properties and corresponding standard deviation across major brain regions for both material models and motion frequency components. Table S2: The mean material properties and corresponding standard deviation across brain regions in subcortical gray matter for both material models and motion frequency components. Table S3: The mean material properties and corresponding standard deviation across brain regions in cortical gray matter for both material models and motion frequency components. Table S4: The mean material properties and corresponding standard deviation across brain regions in white matter tracts for both material models and motion frequency components. Table S5: The mean and standard deviation of the symmetry ratio for all material properties across the major regions for the 1‐Hz motion component. Table S6: The mean and standard deviation of the symmetry ratio for all material properties across the major regions for the 2‐Hz motion component. [file NBM-38-e70073-s001.docx]

# Supplementary material

## 1 Representative parameter maps


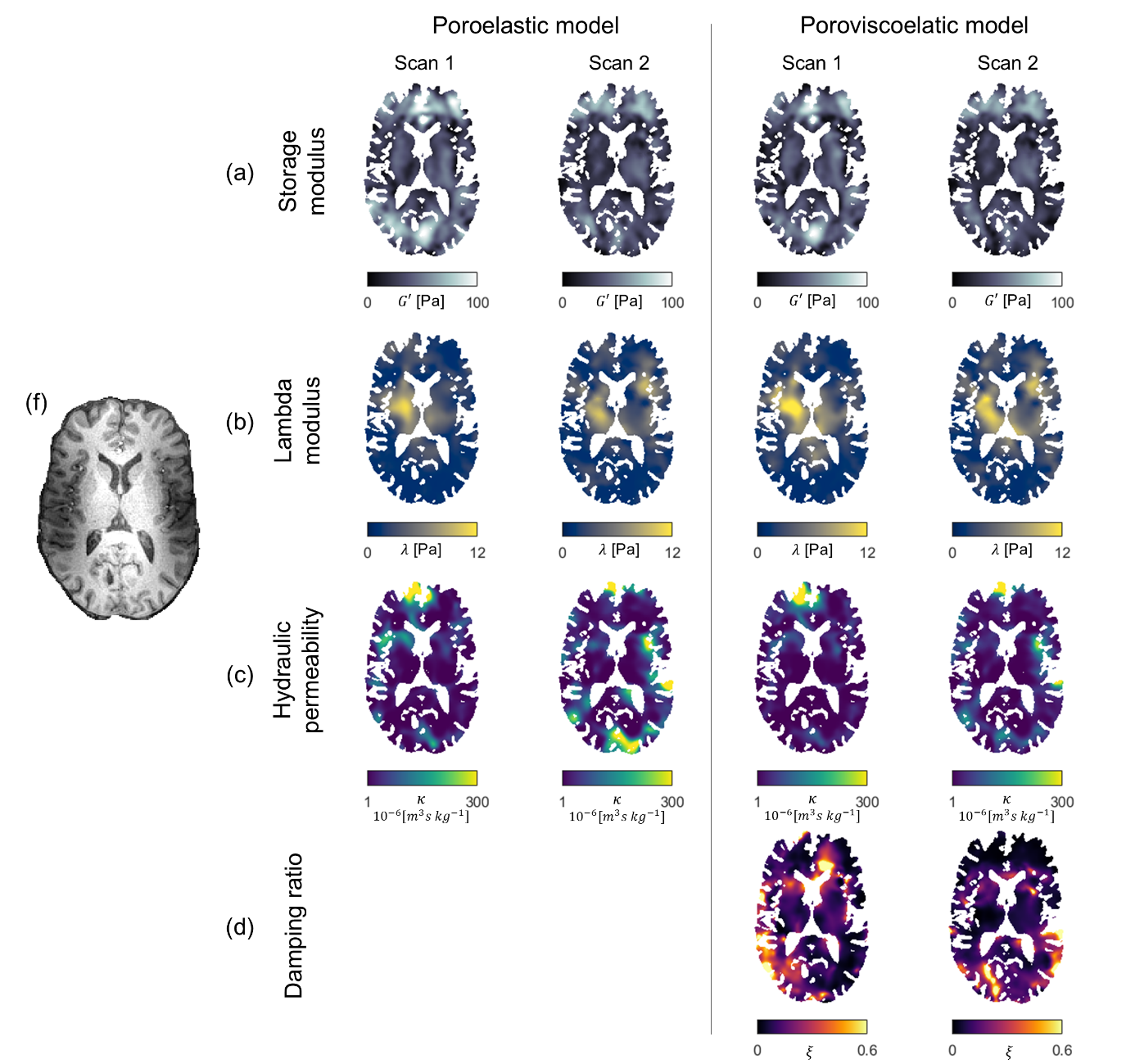


**Figure S1:** Representative axial slices of the storage modulus (a), lambda modulus (b), hydraulic permeability (c), and damping ratio (d) obtained from the 2 Hz motion component for one subject along with a repeated scan which has been co-registered to the first scan. The equivalent slice of the T1-weighted image is shown in (f) for anatomical reference.

## 2 Regional analysis

**Table S1:** The mean material properties and corresponding standard deviation across major brain regions for both material models and motion frequency components.

|  |  | 1 Hz | | 2 Hz | |
| --- | --- | --- | --- | --- | --- |
| Region | **Quantity** | **Poroelastic** | **Poroviscoelastic** | **Poroelastic** | **Poroviscoelastic** |
| Global | $G'$ [Pa] | 6.09 (0.87) | 5.33 (0.77) | 32.08 (11.98) | 26.22 (8.06) |
|  | $\lambda$ [Pa] | 3.99 (0.67) | 4.31 (0.69) | 4.98 (1.60) | 5.58 (1.96) |
|  | $\kappa$ 10^-6^ [m^3^ s kg^-1^] | 266 (79) | 75 (9) | 221 (127) | 52 (17) |
|  | $\xi$ | N/A | 0.23 (0.04) | N/A | 0.27 (0.12) |
| Subcortical GM | $G'$ [Pa] | 4.46 (1.03) | 4.03 (1.23) | 23.88 (11.56) | 20.20 (11.61) |
|  | $\lambda$ [Pa] | 4.00 (0.73) | 4.35 (0.62) | 5.94 (1.82) | 6.49 (2.04) |
|  | $\kappa$ 10^-6^ [m^3^ s kg^-1^] | 419 (260) | 89 (31) | 421 (460) | 60 (43) |
|  | $\xi$ | N/A | 0.31 (0.13) | N/A | 0.48 (0.50) |
| Cortical GM | $G'$ [Pa] | 6.61 (1.18) | 5.58 (1.00) | 35.04 (13.45) | 28.16 (9.01) |
|  | $\lambda$ [Pa] | 3.87 (0.71) | 4.09 (0.72) | 4.49 (1.69) | 4.91 (1.96) |
|  | $\kappa$ 10^-6^ [m^3^ s kg^-1^] | 265 (99) | 78 (22) | 196 (71) | 52 (15) |
|  | $\xi$ | N/A | 0.26 (0.06) | N/A | 0.28 (0.14) |
| WMT | $G'$ [Pa] | 5.84 (1.16) | 4.95 (1.03) | 35.79 (16.39) | 28.57 (12.31) |
|  | $\lambda$ [Pa] | 4.29 (0.60) | 4.76 (0.58) | 5.22 (1.58) | 6.14 (2.13) |
|  | $\kappa$ 10^-6^ [m^3^ s kg^-1^] | 207 (86) | 64 (15) | 140 (111) | 39 (19) |
|  | $\xi$ | N/A | 0.29 (0.09) | N/A | 0.30 (0.17) |

**Table S2:** The mean material properties and corresponding standard deviation across brain regions in subcortical gray matter for both material models and motion frequency components.

|  |  | 1 Hz | | 2 Hz | |
| --- | --- | --- | --- | --- | --- |
| Region | **Quantity** | **Poroelastic** | **Poroviscoelastic** | **Poroelastic** | **Poroviscoelastic** |
| AM | $G'$ [Pa] | 4.38 (1.26) | 4.03 (1.09) | 17.86 (7.91) | 14.66 (6.76) |
| AM | $\lambda$ [Pa] | 2.94 (0.39) | 3.18 (0.54) | 4.05 (1.88) | 4.42 (2.14) |
| AM | $\kappa$ 10^-6^ [m^3^ s kg^-1^] | 141 (67) | 67 (35) | 332 (262) | 55 (22) |
| AM | $\xi$ | N/A | 0.17 (0.04) | N/A | 0.26 (0.10) |
| CA | $G'$ [Pa] | 4.36 (1.00) | 3.92 (1.13) | 24.88 (8.24) | 20.42 (9.14) |
| CA | $\lambda$ [Pa] | 3.46 (0.75) | 3.70 (0.65) | 4.50 (1.18) | 4.51 (0.84) |
| CA | $\kappa$ 10^-6^ [m^3^ s kg^-1^] | 458 (294) | 72 (27) | 194 (254) | 36 (22) |
| CA | $\xi$ | N/A | 0.34 (0.14) | N/A | 0.68 (0.49) |
| HC | $G'$ [Pa] | 3.94 (0.99) | 3.47 (0.94) | 18.21 (6.70) | 15.24 (5.65) |
| HC | $\lambda$ [Pa] | 3.00 (1.03) | 3.27 (1.14) | 4.82 (2.39) | 5.47 (2.59) |
| HC | $\kappa$ 10^-6^ [m^3^ s kg^-1^] | 249 (91) | 88 (72) | 238 (202) | 71 (48) |
| HC | $\xi$ | N/A | 0.23 (0.07) | N/A | 0.25 (0.10) |
| PA | $G'$ [Pa] | 4.61 (0.68) | 4.05 (1.06) | 23.35 (10.63) | 19.41 (11.06) |
| PA | $\lambda$ [Pa] | 4.58 (0.73) | 4.87 (0.81) | 5.32 (1.45) | 5.65 (1.62) |
| PA | $\kappa$ 10^-6^ [m^3^ s kg^-1^] | 451 (219) | 136 (55) | 534 (710) | 64 (64) |
| PA | $\xi$ | N/A | 0.37 (0.24) | N/A | 0.58 (0.48) |
| PU | $G'$ [Pa] | 4.96 (0.66) | 4.58 (1.02) | 28.56 (8.82) | 23.70 (9.73) |
| PU | $\lambda$ [Pa] | 5.34 (0.82) | 5.73 (0.78) | 6.93 (2.69) | 7.09 (2.36) |
| PU | $\kappa$ 10^-6^ [m^3^ s kg^-1^] | 257 (187) | 85 (47) | 212 (284) | 53 (36) |
| PU | $\xi$ | N/A | 0.33 (0.17) | N/A | 0.68 (0.73) |
| TH | $G'$ [Pa] | 4.30 (1.45) | 3.96 (1.59) | 21.94 (14.67) | 19.75 (14.54) |
| TH | $\lambda$ [Pa] | 3.40 (1.00) | 3.84 (1.01) | 5.93 (2.25) | 7.17 (2.32) |
| TH | $\kappa$ 10^-6^ [m^3^ s kg^-1^] | 567 (307) | 89 (33) | 653 (753) | 64 (55) |
| TH | $\xi$ | N/A | 0.27 (0.14) | N/A | 0.31 (0.19) |

**Table S3:** The mean material properties and corresponding standard deviation across brain regions in cortical gray matter for both material models and motion frequency components.

|  |  | 1 Hz | | 2 Hz | |
| --- | --- | --- | --- | --- | --- |
| Region | **Quantity** | **Poroelastic** | **Poroviscoelastic** | **Poroelastic** | **Poroviscoelastic** |
| CN | $G'$ [Pa] | 6.50 (2.11) | 6.07 (2.12) | 39.64 (30.91) | 33.16 (22.02) |
| CN | $\lambda$ [Pa] | 2.31 (1.02) | 2.34 (0.91) | 3.84 (2.93) | 4.08 (3.07) |
| CN | $\kappa$ 10^-6^ [m^3^ s kg^-1^] | 340 (211) | 91 (55) | 176 (136) | 66 (25) |
| CN | $\xi$ | N/A | 0.18 (0.11) | N/A | 0.15 (0.07) |
| FSG | $G'$ [Pa] | 6.13 (0.88) | 5.21 (0.81) | 28.64 (12.30) | 23.53 (7.19) |
| FSG | $\lambda$ [Pa] | 3.34 (0.73) | 3.67 (0.89) | 4.12 (1.64) | 4.81 (1.45) |
| FSG | $\kappa$ 10^-6^ [m^3^ s kg^-1^] | 183 (54) | 93 (26) | 194 (143) | 57 (31) |
| FSG | $\xi$ | N/A | 0.21 (0.07) | N/A | 0.23 (0.10) |
| ITC | $G'$ [Pa] | 5.11 (0.68) | 4.23 (0.49) | 21.05 (4.10) | 17.43 (2.70) |
| ITC | $\lambda$ [Pa] | 2.18 (0.45) | 2.40 (0.52) | 2.29 (0.77) | 2.78 (1.00) |
| ITC | $\kappa$ 10^-6^ [m^3^ s kg^-1^] | 474 (363) | 151 (119) | 376 (169) | 60 (25) |
| ITC | $\xi$ | N/A | 0.24 (0.02) | N/A | 0.30 (0.11) |
| LaO | $G'$ [Pa] | 5.03 (0.65) | 4.68 (0.72) | 33.24 (18.02) | 24.58 (10.17) |
| LaO | lamb $\lambda$ [Pa]da | 3.64 (1.48) | 4.00 (1.57) | 5.75 (3.74) | 6.40 (4.04) |
| LaO | $\kappa$ 10^-6^ [m^3^ s kg^-1^] | 195 (118) | 59 (28) | 152 (173) | 54 (36) |
| LaO | $\xi$ | N/A | 0.18 (0.03) | N/A | 0.45 (0.51) |
| LiO | $G'$ [Pa] | 6.31 (1.36) | 5.55 (1.27) | 32.28 (15.51) | 25.74 (8.04) |
| LiO | $\lambda$ [Pa] | 3.14 (0.99) | 3.19 (0.79) | 4.35 (2.34) | 5.07 (2.49) |
| LiO | $\kappa$ 10^-6^ [m^3^ s kg^-1^] | 244 (230) | 58 (43) | 143 (133) | 41 (16) |
| LiO | $\xi$ | N/A | 0.24 (0.08) | N/A | 0.20 (0.07) |
| PCN | $G'$ [Pa] | 7.45 (2.23) | 6.80 (2.15) | 42.17 (19.05) | 36.13 (15.33) |
| PCN | $\lambda$ [Pa] | 2.44 (0.82) | 2.69 (0.82) | 3.35 (1.57) | 3.77 (1.91) |
| PCN | $\kappa$ 10^-6^ [m^3^ s kg^-1^] | 281 (228) | 67 (23) | 151 (81) | 63 (27) |
| PCN | $\xi$ | N/A | 0.24 (0.17) | N/A | 0.16 (0.06) |
| POST | $G'$ [Pa] | 5.51 (0.96) | 5.22 (0.92) | 28.25 (10.68) | 24.63 (8.27) |
| POST | $\lambda$ [Pa] | 4.41 (1.07) | 4.64 (1.10) | 5.42 (2.61) | 6.39 (2.88) |
| POST | $\kappa$ 10^-6^ [m^3^ s kg^-1^] | 234 (140) | 64 (19) | 154 (72) | 40 (13) |
| POST | $\xi$ | N/A | 0.14 (0.03) | N/A | 0.17 (0.08) |
| PRE | $G'$ [Pa] | 6.56 (1.01) | 6.08 (1.10) | 30.89 (7.88) | 27.52 (6.54) |
| PRE | $\lambda$ [Pa] | 6.19 (1.79) | 6.56 (1.79) | 6.32 (2.18) | 7.28 (2.94) |
| PRE | $\kappa$ 10^-6^ [m^3^ s kg^-1^] | 160 (107) | 60 (24) | 144 (141) | 40 (14) |
| PRE | $\xi$ | N/A | 0.14 (0.04) | N/A | 0.16 (0.06) |
| RMF | $G'$ [Pa] | 8.20 (2.24) | 7.27 (1.89) | 42.39 (15.53) | 34.76 (10.58) |
| RMF | $\lambda$ [Pa] | 5.97 (0.95) | 6.88 (1.15) | 6.30 (2.54) | 6.46 (2.61) |
| RMF | $\kappa$ 10^-6^ [m^3^ s kg^-1^] | 94 (51) | 62 (45) | 77 (46) | 34 (13) |
| RMF | $\xi$ | N/A | 0.17 (0.07) | N/A | 0.33 (0.23) |
| SFC | $G'$ [Pa] | 7.27 (1.63) | 5.18 (1.40) | 37.30 (13.48) | 28.58 (10.39) |
| SFC | $\lambda$ [Pa] | 4.05 (1.07) | 3.94 (1.05) | 3.68 (1.59) | 3.69 (1.52) |
| SFC | $\kappa$ 10^-6^ [m^3^ s kg^-1^] | 351 (174) | 82 (34) | 276 (214) | 58 (34) |
| SFC | $\xi$ | N/A | 0.46 (0.17) | N/A | 0.36 (0.19) |
| SPC | $G'$ [Pa] | 5.91 (1.77) | 5.38 (1.56) | 30.89 (12.62) | 26.20 (9.92) |
| SPC | $\lambda$ [Pa] | 3.21 (0.93) | 3.48 (1.01) | 3.51 (2.05) | 3.90 (2.34) |
| SPC | $\kappa$ 10^-6^ [m^3^ s kg^-1^] | 355 (232) | 61 (25) | 290 (394) | 45 (20) |
| SPC | $\xi$ | N/A | 0.20 (0.07) | N/A | 0.20 (0.09) |
| STC | $G'$ [Pa] | 5.71 (0.54) | 5.11 (0.59) | 27.96 (7.75) | 22.91 (4.74) |
| STC | $\lambda$ [Pa] | 3.60 (0.86) | 3.98 (0.88) | 4.48 (1.60) | 5.17 (2.13) |
| STC | $\kappa$ 10^-6^ [m^3^ s kg^-1^] | 223 (132) | 79 (28) | 130 (73) | 43 (17) |
| STC | $\xi$ | N/A | 0.18 (0.04) | N/A | 0.24 (0.08) |

**Table S4:** The mean material properties and corresponding standard deviation across brain regions in white matter tracts for both material models and motion frequency components.

|  |  | 1 Hz | | 2Hz | |
| --- | --- | --- | --- | --- | --- |
| Region | **Field** | **Poroelastic** | **Poroviscoelastic** | **Poroelastic** | **Poroviscoelastic** |
| ATR | $G'$ [Pa] | 5.24 (1.30) | 4.70 (1.41) | 26.48 (11.74) | 21.85 (11.29) |
| ATR | $\lambda$ [Pa] | 4.19 (0.80) | 4.81 (0.81) | 5.89 (1.80) | 6.90 (1.97) |
| ATR | $\kappa$ 10^-6^ [m^3^ s kg^-1^] | 338 (205) | 73 (19) | 537 (599) | 50 (36) |
| ATR | $\xi$ | N/A | 0.27 (0.09) | N/A | 0.44 (0.29) |
| CC | $G'$ [Pa] | 5.81 (1.26) | 4.81 (1.46) | 32.39 (12.66) | 26.39 (10.32) |
| CC | $\lambda$ [Pa] | 3.11 (0.38) | 3.29 (0.34) | 3.92 (0.94) | 4.57 (1.14) |
| CC | $\kappa$ 10^-6^ [m^3^ s kg^-1^] | 318 (159) | 76 (34) | 87 (63) | 34 (22) |
| CC | $\xi$ | N/A | 0.45 (0.15) | N/A | 0.34 (0.13) |
| CRa | $G'$ [Pa] | 5.75 (1.36) | 5.03 (1.06) | 36.86 (16.60) | 29.88 (12.88) |
| CRa | $\lambda$ [Pa] | 5.12 (0.64) | 6.00 (0.71) | 6.10 (2.45) | 7.62 (3.39) |
| CRa | $\kappa$ 10^-6^ [m^3^ s kg^-1^] | 110 (29) | 40 (7) | 69 (61) | 29 (18) |
| CRa | $\xi$ | N/A | 0.24 (0.04) | N/A | 0.28 (0.12) |
| CST | $G'$ [Pa] | 4.28 (0.97) | 4.18 (0.93) | 26.94 (15.03) | 23.95 (12.78) |
| CST | $\lambda$ [Pa] | 4.18 (1.02) | 4.78 (1.05) | 6.89 (3.17) | 7.91 (3.30) |
| CST | $\kappa$ 10^-6^ [m^3^ s kg^-1^] | 234 (125) | 59 (22) | 382 (470) | 60 (48) |
| CST | $\xi$ | N/A | 0.18 (0.06) | N/A | 0.21 (0.10) |
| FMa | $G'$ [Pa] | 5.86 (1.54) | 5.25 (1.52) | 39.07 (27.14) | 30.84 (15.85) |
| FMa | $\lambda$ [Pa] | 2.67 (0.77) | 3.16 (0.89) | 4.30 (1.97) | 5.63 (2.23) |
| FMa | $\kappa$ 10^-6^ [m^3^ s kg^-1^] | 171 (107) | 44 (27) | 47 (30) | 26 (15) |
| FMa | $\xi$ | N/A | 0.24 (0.11) | N/A | 0.18 (0.08) |
| FMi | $G'$ [Pa] | 7.15 (2.26) | 4.75 (1.13) | 41.16 (16.73) | 28.34 (11.58) |
| FMi | $\lambda$ [Pa] | 4.29 (0.88) | 4.49 (0.92) | 4.03 (1.60) | 3.98 (1.76) |
| FMi | $\kappa$ 10^-6^ [m^3^ s kg^-1^] | 252 (154) | 63 (11) | 122 (51) | 48 (26) |
| FMi | $\xi$ | N/A | 0.50 (0.25) | N/A | 0.49 (0.27) |
| IFOF | $G'$ [Pa] | 6.42 (0.71) | 5.83 (0.66) | 35.38 (12.77) | 27.32 (7.34) |
| IFOF | $\lambda$ [Pa] | 4.48 (1.21) | 5.04 (1.21) | 5.48 (1.70) | 6.29 (2.43) |
| IFOF | $\kappa$ 10^-6^ [m^3^ s kg^-1^] | 119 (26) | 60 (15) | 80 (58) | 41 (19) |
| IFOF | $\xi$ | N/A | 0.20 (0.07) | N/A | 0.34 (0.18) |
| ILF | $G'$ [Pa] | 6.05 (0.97) | 5.33 (0.81) | 28.54 (8.52) | 23.37 (5.22) |
| ILF | $\lambda$ [Pa] | 3.32 (0.98) | 3.57 (0.99) | 3.45 (1.38) | 4.13 (1.61) |
| ILF | $\kappa$ 10^-6^ [m^3^ s kg^-1^] | 152 (102) | 71 (36) | 103 (76) | 41 (17) |
| ILF | $\xi$ | N/A | 0.18 (0.05) | N/A | 0.21 (0.07) |
| PTR | $G'$ [Pa] | 6.27 (1.22) | 5.44 (0.96) | 39.08 (27.55) | 29.25 (13.66) |
| PTR | $\lambda$ [Pa] | 3.28 (1.35) | 3.80 (1.43) | 4.15 (2.48) | 5.13 (3.14) |
| PTR | $\kappa$ 10^-6^ [m^3^ s kg^-1^] | 146 (64) | 73 (44) | 45 (25) | 34 (21) |
| PTR | $\xi$ | N/A | 0.18 (0.04) | N/A | 0.22 (0.11) |
| SLF | $G'$ [Pa] | 4.98 (0.65) | 4.67 (0.62) | 36.00 (19.91) | 31.34 (14.96) |
| SLF | $\lambda$ [Pa] | 5.21 (1.15) | 5.70 (1.08) | 6.01 (2.81) | 7.29 (3.56) |
| SLF | $\kappa$ 10^-6^ [m^3^ s kg^-1^] | 145 (96) | 74 (54) | 53 (39) | 28 (16) |
| SLF | $\xi$ | N/A | 0.16 (0.04) | N/A | 0.18 (0.08) |

## 3 Symmetry analysis

**Table S5:** The mean and standard deviation of the symmetry ratio for all material properties across the major regions for the 1 Hz motion component.

|  | Region | Poroelastic | Poroviscoelastic |
| --- | --- | --- | --- |
| Storage modulus | Global | 0.99 (0.05) | 0.99 (0.04) |
|  | SGM | 0.99 (0.18) | 0.96 (0.14) |
|  | CGM | 1.01 (0.06) | 1.01 (0.04) |
|  | WMT | 1.02 (0.11) | 1.05 (0.12) |
| Lambda modulus | Global | 0.93 (0.14) | 0.89 (0.13) |
|  | SGM | 0.89 (0.29) | 0.79 (0.24) |
|  | CGM | 1.00 (0.20) | 0.95 (0.20) |
|  | WMT | 1.09 (0.21) | 0.97 (0.19) |
| Hydraulic permeability | Global | 1.20 (0.30) | 1.37 (0.25) |
|  | SGM | 3.34 (2.97) | 3.59 (2.78) |
|  | CGM | 1.23 (0.33) | 1.52 (0.44) |
|  | WMT | 1.42 (0.31) | 1.29 (0.41) |
| Damping ratio | Global | N/A | 0.94 (0.10) |
|  | SGM | N/A | 1.14 (0.19) |
|  | CGM | N/A | 0.95 (0.16) |
|  | WMT | N/A | 0.90 (0.17) |

**Table S6:** The mean and standard deviation of the symmetry ratio for all material properties across the major regions for the 2 Hz motion component.

|  | Region | Poroelastic | Poroviscoelastic |
| --- | --- | --- | --- |
| Storage modulus | Global | 1.02 (0.06) | 1.06 (0.13) |
|  | SGM | 1.03 (0.21) | 0.95 (0.15) |
|  | CGM | 1.04 (0.09) | 1.09 (0.15) |
|  | WMT | 1.02 (0.11) | 1.03 (0.14) |
| Lambda modulus | Global | 0.99 (0.23) | 0.98 (0.26) |
|  | SGM | 1.22 (0.35) | 1.03 (0.37) |
|  | CGM | 1.02 (0.41) | 0.99 (0.41) |
|  | WMT | 1.18 (0.25) | 1.12 (0.34) |
| Hydraulic permeability | Global | 1.13 (0.70) | 1.22 (0.37) |
|  | SGM | 1.42 (0.43) | 1.89 (0.66) |
|  | CGM | 1.08 (0.79) | 1.25 (0.51) |
|  | WMT | 1.45 (0.87) | 1.37 (0.45) |
| Damping ratio | Global | N/A | 0.87 (0.20) |
|  | SGM | N/A | 1.62 (0.52) |
|  | CGM | N/A | 0.80 (0.24) |
|  | WMT | N/A | 1.06 (0.36) |
